# Multiple imputation for cause-specific Cox models: Assessing methods for estimation and prediction

**DOI:** 10.1177/09622802221102623

**Published:** 2022-06-05

**Authors:** Edouard F Bonneville, Matthieu Resche-Rigon, Johannes Schetelig, Hein Putter, Liesbeth C de Wreede

**Affiliations:** 1 Department of Biomedical Data Sciences, 4501 Leiden University Medical Center, Leiden, The Netherlands; 2 Service de Biostatistique et Information Médicale, 55663 Hôpital Saint-Louis, Paris, France; 3 538360 Centre de Recherche en Epidémiologie et Statistiques Sorbonne Paris Cité, Paris, France; 4 ECSTRRA Team, 27102 INSERM, Paris, France; 5 39063 Dresden University Hospital, Dresden, Germany; 6 DKMS Clinical Trials Unit, Dresden, Germany

**Keywords:** Competing risks, cause-specific hazards, multiple imputation, missing covariates, substantive model compatible imputation, Cox model

## Abstract

In studies analyzing competing time-to-event outcomes, interest often lies in both estimating the effects of baseline covariates on the cause-specific hazards and predicting cumulative incidence functions. When missing values occur in these baseline covariates, they may be discarded as part of a complete-case analysis or multiply imputed. In the latter case, the imputations may be performed either compatibly with a substantive model pre-specified as a cause-specific Cox model [substantive model compatible fully conditional specification (SMC-FCS)], or approximately so [multivariate imputation by chained equations (MICE)]. In a large simulation study, we assessed the performance of these three different methods in terms of estimating cause-specific regression coefficients and predicting cumulative incidence functions. Concerning regression coefficients, results provide further support for use of SMC-FCS over MICE, particularly when covariate effects are large and the baseline hazards of the competing events are substantially different. Complete-case analysis also shows adequate performance in settings where missingness is not outcome dependent. With regard to cumulative incidence prediction, SMC-FCS and MICE are performed more similarly, as also evidenced in the illustrative analysis of competing outcomes following a hematopoietic stem cell transplantation. The findings are discussed alongside recommendations for practising statisticians.

## 1 Introduction

Missing covariate data are of perennial concern in observational studies in medicine.^1^ The backbone of such studies are clinical registries, which collect patient data potentially spanning many countries and centres over long periods of time. These and other data management complexities can lead to various patterns of (possibly informative) missingness. Furthermore, these registries are often set up for multiple purposes leading to multiple studies where different potentially exclusive survival outcomes could be considered. Consequently, *competing risks* outcomes are frequently investigated. This refers to a setting in which individuals can only experience one of several mutually exclusive events.

In studies considering competing risk outcomes, interest can lie in both the probabilities of events occurring over time and the effect of covariates on the different competing events. Appropriate handling of missing data is then of central concern in view of avoiding potential bias and/or loss of power when estimating these quantities, as could be expected when using simple methods such as complete-case analysis (CCA).^2^

A more principled approach to handling missing covariate data is to use multiple imputation (MI), where a set of complete data sets is generated using samples based on an imputation model to fill in the missing values.^3^ A substantive model is then run on each of these data sets, before combining the estimates using rules that adequately reflect the uncertainty in the imputation procedure.^4^ The imputation model and the substantive model should ideally be compatible, that is, deriving from a joint model under which both models are conditionals. If data are missing across multiple covariates, the fully conditional specification approach can be used.^5^ This involves specifying an imputation model for each variable with missing values, fully conditional on the other variables, including the outcome. The procedure is better known under its more popular name ‘multivariate imputation by chained equations’ (MICE).^6^

In time-to-event analysis, a popular choice of substantive model is the Cox proportional hazards model. White and Royston^7^ showed that when using MICE in the context of a Cox model (in absence of competing events), for each covariate with missing data, the corresponding imputation model should include the remaining covariates, the event indicator, and the cumulative baseline hazard. To implement this model, the cumulative baseline hazard can be approximated by the marginal Nelson–Aalen estimate of the cumulative hazard. Moreover, depending on the type of covariate, the imputation model is simplified with a Taylor approximation for the non-linear terms from the Cox likelihood. In view of this approximate compatibility between the substantive and imputation model, Bartlett et al.^8^ proposed a variant of MICE called ‘substantive model compatible fully conditional specification’ (SMC-FCS). The approach ensures full compatibility between the imputation model and the substantive model by imputing missing covariate values in a rejection sampling procedure.

In competing risk settings, where the analysis model of interest is often a *cause-specific* Cox proportional hazards model, there has been little research addressing the appropriate use of MI when imputing missing covariate data.^9^ The most prominent work is that of Bartlett and Taylor, where the SMC-FCS approach was extended for cause-specific Cox models.^10^ In a simulation study as part of their work, Bartlett and Taylor compared SMC-FCS to an approximate MICE procedure proposed by Resche-Rigon et al.^11^ The proposal was an extension of the work of White and Royston for cause-specific Cox models. Simulation results suggested using SMC-FCS generally leads to estimates with little bias and nominal coverage.^10^ In contrast, the approximate MICE approach was often biased, with some mitigation using interaction terms in the imputation model.

Importantly, we remark that the algebraic motivation behind the approximate MICE approach is currently unpublished. Moreover, the work of Bartlett and Taylor is to our knowledge the only empirical comparison of this approximate MICE approach with the SMC-FCS approach. Thus, questions regarding the performance of both methods in a wider range of situations still remain. In addition, the question of how both the approaches perform with regard to predicted cumulative incidence functions is hitherto unexplored.

The aim of the present research is thus threefold. First, we aim to formally extend the work of White and Royston for cause-specific Cox models. Specifically, we will derive the approximately compatible imputation models for continuous, binary and multi-level categorical missing covariates. This extension was originally initiated by one of the authors of the current manuscript and shared as part of an oral presentation.^11^ Second, we aim to replicate and extend the simulations of Bartlett and Taylor; additionally manipulating the shape of the competing baseline hazards and the strength of missingness mechanisms, among other extensions. Third, we will explore how biases in cause-specific Cox models affect predicted cumulative incidence functions for patterns of reference covariate values. Simulation results will be interpreted alongside an illustrative analysis using a data set from the field of allogeneic hematopoietic stem cell transplantation (alloHCT).

In the Section 2, we present the motivating data set, and in the Section 3 we introduce notation for cause-specific competing risks analysis. In the Section 4.1 section, the algebraic motivation behind the imputation model for a cause-specific Cox analysis model is shown. The simulation study is presented in the Section 5, followed by an illustrative analysis in the Section 6. Findings are discussed alongside recommendations for practice in the Section 7.

## 2 Motivating example

Schetelig et al.^12^ assessed long-term outcomes of patients with myelodysplastic syndromes (MDS) or secondary acute myeloid leukemia (sAML) after an alloHCT. MDS is characterised by the production of deficient clonal blood cells in the bone marrow and can rapidly progress to more severe sAML.^13^ AlloHCT is the only treatment that can offer long-term remission of the disease. Therefore, alloHCT is recommended for disease stages at high risk of transformation into acute myeloid leukemia (AML) or death from other complications. However, this procedure is associated with a high risk of adverse outcomes, either due to relapse of MDS or sAML, or due to side effects of the (pre-)treatment. This leads to the competing risks outcomes relapse and non-relapse mortality.

The data set contains 6434 patients transplanted between 2000 and 2012, and registered with the European Society for Blood and Marrow Transplantation (EBMT). Several possible predictors measured at the time of transplantation have a substantial amount of missing values. Some examples of variables with missing values are cytogenetic classification (62.2% missing), comorbidity index (59.9% missing) and the Karnofsky performance score (32.8% missing). A cause-specific model for relapse with the aforementioned three variables as predictors, performed on complete cases only, makes use of a mere 20% of the full data set. The immediate lack of efficiency here prompted an investigation as to the performance of MI for such examples.

## 3 Cause-specific competing risks analysis

In a competing risks setting, we assume that individuals can ‘fail’ from only one of K distinct events. We denote that failure time as $\tilde{T}$ , and the competing event indicator as $\tilde{D}\in \{1,\ldots ,K\}$ . In practice, individuals are subject to some right-censoring time C , which is assumed to be independent of $\tilde{T}$ and $\tilde{D}$ , possibly given covariates. We thus only observe realisations $\left(t_{i},d_{i}\right)$ of $T=\min(C,\tilde{T})$ and $D=I(\tilde{T}\leq C)\tilde{D}$ , where D=0 indicates a right-censored observation.

If we view competing risks as a multi-state process, with a single (event-free) initial state and K absorbing states, interest often lies in the cause-specific hazard, defined for a single event k as

$$h_{k}(t)=\lim_{\Delta t\to 0}\frac{P(t\leq \tilde{T}<t+\Delta t,\tilde{D}=k|\tilde{T}\geq t)}{\Delta t}.$$

This hazard function can be interpreted as the instantaneous force of transition, or intensity, of moving between the initial state and state k .^14,15^ A model can then be specified, conditional on a covariate vector Z . A Cox model is a common choice, defined for a failure cause k as

$$h_{k}(t|Z)=h_{k0}(t)\exp\;(\beta_{k}^{T}Z),$$

where $h_{k0}(t)$ is the cause-specific baseline hazard, and $\beta_{k}$ represents the effects of covariates Z on the cause-specific hazard. We note that in what follows, we use ‘effect’ to refer to the impact of a covariate in a multivariable model where there may be non-negligible additional confounding, and this should hence not be interpreted as a fully causal quantity. Furthermore, the K hazard functions define the failure-free survival probability:

$$S(t|Z)=\exp\;\left(-\sum_{k=1}^{K}\int_{0}^{t}h_{k}(u|Z)du\right)=\exp\;\left(-\sum_{k=1}^{K}H_{k}(t|Z)\right),$$

where $H_{k}(t|Z)=\int_{0}^{t}h_{k}(u|Z)du$ is the cause-specific cumulative hazard for cause k . Assuming conditional non-informative censoring, the likelihood contribution of an individual with observations $\left(t_{i},d_{i},z_{i}\right)$ is then

(1) $$p(t_{i},d_{i}|z_{i})=S(t_{i}|z_{i})\prod_{k=1}^{K}{\left[h_{k}(t_{i}|z_{i})\right]}^{I(d_{i}=k)},$$

where I(⋅) is the indicator function. The covariate effects $\beta_{k}$ on the cause-specific hazard can then be estimated by optimising the partial likelihood.^16^ This follows from the observation that the above expression factorises into separate factors for each cause k , which each corresponding to a standard Cox likelihood function where events from all other causes are treated as censored observations.^17^

### 3.1 Cumulative incidence functions

Beyond assessing covariates, cause-specific hazards can also be used to estimate the so-called cumulative incidence functions, defined as

(2) $$P(\tilde{T}\leq t,\tilde{D}=k)=\int_{0}^{t}h_{k}(u)S(u-)\;du,\;k=1,\ldots ,K,$$

where S(u−) is the failure-free survival probability just prior to u .^18^ This cumulative incidence function, or transition probability, is the probability of experiencing event k before or at time t . It is also known as the absolute, or crude risk. It can be computed either non-parametrically, or semi-parametrically if Cox models are specified for the $h_{k}(u)$ . In the latter case, the cumulative hazards derived from the Breslow estimator of the cumulative cause-specific baseline hazards are used as ingredients for estimating the cumulative incidence for cause k .

This implies that we do not need to model the cumulative incidence function *directly* in order to obtain these predicted probabilities, as is done when using the Fine-Gray model.^19^ This is helpful given that in observational studies, interest is seldom in prediction alone: predictions are often presented after first reporting and interpreting model coefficients. The cause-specific hazards framework provides a more natural scale on which to interpret covariate effects and allows to obtain predicted patient-specific cumulative incidence functions for all causes.

## 4 Methods

In this section, we provide a framework for using MICE and SMC-FCS for both estimation of cause-specific regression coefficients and cumulative incidence functions. Throughout, we assume that data are missing according to a missing (completely) at random mechanism, hereafter abbreviated as M(C)AR.

### 4.1 Fully conditional approach (MICE)

We introduce X as a single, partially observed covariate, and Z as a fully observed covariate. We note that Z could also represent a vector of complete covariates. Appropriate use of MICE for cause-specific competing risks analysis requires the specification of an *imputation model* p(X∣T,D,Z) , from which a number of imputed data sets are generated. Detailed derivations for p(X∣T,D,Z) are provided in appendix A, which we summarise in the present subsection.

To begin with, we note that by Bayes’ Theorem,

(3) logp(X∣T,D,Z)=logp(T,D∣X,Z)+logp(X∣Z)+c,

where c is a constant term that does not depend on X . For p(T,D∣X,Z) , a cause-specific Cox proportional hazards model for each failure cause k is specified as $h_{k}(t|X,Z)=h_{k0}(t)\exp\;(\beta_{k}X+\gamma_{k}Z)$ . In case of binary or continuous X and Z , $\beta_{k}$ and $\gamma_{k}$ are scalars; for categorical X or Z with two or more levels, $\beta_{k}$ and $\gamma_{k}$ are vectors and X and Z represent dummy codings for the levels of the covariates. To impute from the fully conditional distribution in Equation (3), we also need to specify a model for the missing data, p(X∣Z) . This model will generally vary depending on the covariate type of X .

#### 4.1.1 Binary X

If X is binary, we could assume $\mathrm{logit}\;P(X=1|Z)=\zeta_{0}+\zeta_{1}Z$ . If Z is categorical with J≥2 levels (without loss of generality assuming that Z takes values in 1,…,J ), we can write

(4) $$\begin{array}{rl} \mathrm{logit}\;P(X=1|T,D,Z) & =\alpha_{0}+\sum_{k=1}^{K}\alpha_{k}I(D=k)+\sum_{k=1}^{K}\alpha_{K+k}H_{k0}(T)\\ & \;+\sum_{\;j=1}^{J-1}\alpha_{2K+j}I(Z=j)+\sum_{\;j=1}^{J-1}\sum_{k=1}^{K}\alpha_{(j+1)K+(J-1)+k}I(Z=j)H_{k0}(T), \end{array}$$

which implies that for categorical Z we can impute missing X values using a logistic regression with D (as a factor variable), the cumulative baseline hazards for all causes of failure, Z (as a factor variable), and the complete interactions between the cumulative baseline hazards and Z . For continuous Z , results are no longer exact. Using a first-order Taylor approximation for the $\exp\;(\gamma_{k}Z)$ term, we can write

(5) $$\begin{array}{rl} \mathrm{logit}\;P(X=1|T,D,Z) & \approx \alpha_{0}+\sum_{k=1}^{K}\alpha_{k}I(D=k)+\sum_{k=1}^{K}\alpha_{K+k}H_{k0}(T)+\sum_{k=1}^{K}\alpha_{2K+k}H_{k0}(T)Z\\ & \;+\alpha_{3K+1}Z, \end{array}$$

which is valid if $\mathrm{Var}(\gamma_{k}Z)$ is small. This approximate imputation model thus uses D , Z , all $H_{k0}(T)$ and the interactions between all $H_{k0}(T)$ and Z as predictors in a logistic regression. Note that the α parameters used above and in the next subsections represent the imputation model coefficients, and are themselves functions of other (substantive and missing data model) parameters. Therefore, these will vary depending on the covariate types of X and Z , and the parametrisation of the substantive model (i.e. whether each cause-specific model has the same predictors, and their functional forms).

#### 4.1.2 Nominal categorical X

If X is a categorical covariate with J≥2 levels and j={0,…,J−1} , we can specify different imputation models depending on whether X is ordered or not. In the unordered (nominal) case, we can specify a multinomial logistic regression for p(X∣Z) , yielding

(6) $$\begin{array}{rl} \log\;\frac{P(X=j|T,D,Z)}{P(X=0|T,D,Z)} & \approx \alpha_{\;j,0}+\sum_{k=1}^{K}\alpha_{\;j,k}I(D=k)+\sum_{k=1}^{K}\alpha_{\;j,K+k}H_{k0}(T)+\sum_{k=1}^{K}\alpha_{\;j,2K+k}H_{k0}(T)Z\\ & \;+\alpha_{\;j,3K+1}Z. \end{array}$$

This comes as a result of generalising $\mathrm{logit}\;P(X=1|Z)=\zeta_{0}+\zeta_{1}Z$ to $\log\;\frac{P(X=j|Z)}{P(X=0|Z)}=\zeta_{0}+\zeta_{j}Z$ , and holds for continuous Z as in (5). For categorical or no Z , where for the former I(Z=j) should be used as in equation (4), the expression for the fully conditional distribution is exact as in the binary case. The predictors to be included in the imputation model are exactly the same as for binary X .

#### 4.1.3 Ordered categorical X

For ordered categorical X , a proportional odds model could be assumed as $\mathrm{logit}\;P(X\leq j|Z)=\zeta_{\;j}+\zeta_{Z}Z$ . This however implies that the fully conditional distribution requires specifying p(T,D∣X≤j,Z) , which does not have a standard proportional hazards density. Instead, it has a *weighted sum* of proportional hazards densities. Thus, the expression for P(X≤j∣T,D,Z) does not extend from the binary case in any simple form. Nevertheless, a proportional odds model including D , Z and all $H_{k0}(T)$ could still be used to impute the missing X values, though the properties of such a model are not currently well known. We refer the reader to the book written by McCullagh and Nelder for a detailed description of both the multinomial logistic regression and proportional odds models.^20^

#### 4.1.4 Continuous X

If X is a continuous covariate, we could assume it to be normal conditional on Z (possibly after transformation), as $X|Z∼N(\zeta_{0}+\zeta_{1}Z,\sigma^{2})$ . The implied expression for p(X∣T,D,Z) is not normal due to the $\exp\;(\beta_{k}X+\gamma_{k}Z)$ term, and so a bivariate Taylor approximation is used around the sample means $\bar{X}$ and $\bar{Z}$ . To the first degree, the approximate fully conditional density is expressed as

$$X|T,D,Z∼N(\alpha_{0}+\alpha_{1}Z+\sum_{k=1}^{K}\alpha_{k+1}I(D=k)+\sum_{k=1}^{K}\alpha_{K+k+1}H_{k0}(T),\sigma^{2}).$$

This suggests a model for imputing continuous X should be a linear regression with D , Z and all $H_{k0}(T)$ again as predictors. With a quadratic approximation for $\exp\;(\beta_{k}X+\gamma_{k}Z)$ , the accuracy of the above model can be improved by additionally including the interactions between all $H_{k0}(T)$ and Z . The approximations are valid under the assumption of small $\mathrm{Var}(\beta_{k}X+\gamma_{k}Z)$ .

We note that the above models, like in the simple time-to-event settings, cannot be implemented without a working estimate of $H_{k0}(T)$ – whose true values we will assume are unknown. For the competing risks setting, we can use the marginal Nelson–Aalen estimate of the cumulative cause-specific hazard (which requires treating all events other than k as censored) as an approximation for $H_{k0}(T)$ . As explained by White and Royston, this approximation becomes poorer with larger true covariate effects.^7^ We may then expect the estimated covariate effects after the imputation procedure to be biased.

### 4.2 Substantive model compatible approach

We refer the reader to the work of Bartlett et al.^8^ for a detailed introduction of the SMC-FCS method, and to the work of Bartlett and Taylor^10^ for its specific extension to cause-specific Cox proportional hazards models. Briefly, the SMC-FCS method (in the current setting) is based on the application of Bayes’ theorem,

(7) p(X∣T,D,Z)∝p(T,D∣X,Z)p(X∣Z),

which was already introduced on the logarithmic scale in Equation (3). The parameters associated with both p(T,D∣X,Z) and p(X∣Z) are omitted for readability. In essence, the procedure involves choosing p(X∣Z) as a proposal density and using rejection sampling to draw possible values for missing X from a density proportional to p(T,D∣X,Z)p(X∣Z) . This is under the assumption that p(X∣Z) is simple to sample from, as is the case if we specify a model for it, e.g. a linear regression of X conditional on Z . The imputation model is then compatible with the substantive model in the sense that a joint distribution exists which contains both the substantive model and the imputation model as its conditional distributions. If multiple covariates have missing data, it is still possible to specify mutually incompatible models for p(X∣Z) , but each fully conditional distribution will be compatible with the substantive model.

In contrast to MICE, the SMC-FCS approach does not require any approximations – neither for the non-linear terms nor for the cumulative baseline hazard. Of course, the cumulative baseline hazard still needs to be evaluated in order to draw from (7). In order to do so, the Breslow estimate is used and is updated at each iteration of the imputation procedure conditional on the most recent draws from the posterior distribution of the regression coefficients.

### 4.3 Regression coefficients

Both the MICE and SMC-FCS procedures result in m=1,…,M imputed data sets. In each of these data sets, the cause-specific Cox model for one or more of the K causes of failure is fitted. Let θ denote a cause-specific regression coefficient of interest, and let ${\hat{\theta }}_{m}$ and $\hat{\mathrm{Var}}({\hat{\theta }}_{m})$ , respectively denote the estimate and associated variance of this coefficient in the m th imputed data set. We can combine these M estimates using Rubin’s rules, with estimator

$$\hat{\theta }=\frac{1}{M}\sum_{m=1}^{M}{\hat{\theta }}_{m}.$$

The associated variance estimator is

$$\hat{\mathrm{Var}}(\hat{\theta })=\frac{1}{M}\sum_{m=1}^{M}\hat{\mathrm{Var}}({\hat{\theta }}_{m})+(1+\frac{1}{M})\frac{1}{M-1}\sum_{m=1}^{M}({\hat{\theta }}_{m}-\hat{\theta }{)}^{2},$$

which combines estimates of within and between imputation variance.^4^ The estimate of the standard error is then readily obtained as $\hat{\mathrm{SE}}(\hat{\theta })=\sqrt{\hat{\mathrm{Var}}(\hat{\theta })}$ .

### 4.4 Predicted probabilities

To obtain the predicted cumulative incidence functions for an individual with fully observed covariates after an MI procedure, there are at least two possible options. The first is to pool the regression coefficients and baseline hazards separately, and use those to produce a single predicted curve. The second approach is to use the substantive models fitted in each imputed data set to create *imputation-specific* predictions, and then pool those (possibly after transformation) using Rubin’s rules. The articles by Wood et al.^21^ and Mertens et al.^22,23^ recommend the second approach, which is the one we employ in the present paper.

## 5 Simulation study

We designed a simulation study with the aim of comparing the performance of CCA, MICE and SMC-FCS in the presence of missing baseline covariate values for cause-specific Cox proportional hazards models with two competing events. We assessed performance with respect to estimated regression coefficients and predicted cumulative incidence functions.

### 5.1 Data-generating mechanisms

We generated data sets containing n=2000 individuals, with one record each containing both predictor and outcome information.

#### 5.1.1 Covariates

Two covariates X and Z were generated in each data set. We varied the covariate type of X as either continuous or binary, and Z was fixed as continuous. When both covariates were continuous, they were generated from a bivariate standard normal distribution X,Z∼N(μ,Σ) , with means μ={0,0} , variances diag(Σ)={1,1} and correlation ρ=0.5 .

When X was binary, we assumed X∼Bern(0.5) and Z∼N(0,1) , with a *point-biserial* correlation between the two variables of ρ=0.5 . We can generate observations in this way by first generating $X^{\prime }$ and Z from a bivariate standard normal distribution with correlation $\rho^{\prime }\approx 0.63$ , and then dichotomising $X^{\prime }$ at 0 (the value of the standard normal quantile function for a probability of 0.5) to produce X . We refer the reader to the work of Demirtas and Hedeker for a description of this well-established procedure.^24^

#### 5.1.2 Competing event times

We based our simulation of event times on the motivating alloHCT example described in the Section 2 section, focusing on the two competing events relapse (REL) and non-relapse mortality (NRM) over a 10-year follow-up period. To generate the failure times for the competing events, we made use of latent failure times, denoted ${\tilde{T}}_{1}\;and\;{\tilde{T}}_{2}$ for REL and NRM, respectively.^25^

Typically in alloHCT studies, patients are at very high risk of both relapse and NRM in the initial period after alloHCT, with this risk gradually decreasing thereafter as they survive longer. For this reason, generating failure times from a distribution with a decreasing hazard function is appropriate. The Weibull distribution, with probability density function $f(t)=\kappa \lambda t^{\kappa -1}\exp\;(-\lambda t^{\kappa })$ , with shape κ>0 and rate λ>0 , accommodates decreasing hazards for κ<1 . This is the parametrisation used in the text by Klein and Moeschberger.^26^

We thus generated both latent failure times from independent Weibull distributions, assuming cause-specific proportional hazards conditional on X and Z . We furthermore generated independent censoring times from an Exponential distribution. In summary:

$$\begin{array}{rl} {\tilde{T}}_{1} & ∼\text{Weibull}(\kappa_{1},\lambda_{1}=\lambda_{10}e^{\beta_{1}X+\gamma_{1}Z}),\\ {\tilde{T}}_{2} & ∼\text{Weibull}(\kappa_{2},\lambda_{2}=\lambda_{20}e^{\beta_{2}X+\gamma_{2}Z}),\\ C & ∼\text{Exp}(\lambda_{C}), \end{array}$$

where $\lambda_{C}$ is the censoring rate, and $\lambda_{10}$ and $\lambda_{20}$ are the baseline hazard rates for REL and NRM, respectively. We then defined $\tilde{T}=\text{m}in({\tilde{T}}_{1},{\tilde{T}}_{2})$ , with an associated factor variable $\tilde{D}$ , where $\tilde{D}=1$ if REL occured first, and $\tilde{D}=2$ otherwise. The generated observed (event or censoring) time was then defined as $T=\text{m}in(C,\tilde{T})$ , with corresponding indicator $D=I(\tilde{T}\leq C)\tilde{D}$ .

We used estimates from cause-specific marginal accelerated failure time (AFT) models on the motivating data set to fix the parameters values of the baseline shape and hazard rates for the latent failure times. Weibull AFT models for both causes of failure led to fixing $\kappa_{1}=0.58$ , $\lambda_{10}=0.19$ , $\kappa_{2}=0.53$ , and $\lambda_{20}=0.21$ . An exponential AFT model for the censoring distribution motivated setting $\lambda_{C}=0.14$ . Since the baseline hazards for both competing events were estimated to be very similar, we decide to also vary $\left\{\kappa_{1},\lambda_{10}\}=\{1.5,0.04\right\}$ , such that REL had a steadily increasing hazard. Both these ‘similar’ and ‘different’ baseline hazard configurations lead to comparable marginal 10-year cumulative incidences of both events, in the 35–45% range. Regarding cause-specific regression coefficients, we varied $\beta_{1}=\{0,0.5,1\}$ , and fixed $\gamma_{1}=1$ , $\beta_{2}=0.5$ and $\gamma_{2}=0.5$ .

#### 5.1.3 Missing data mechanisms

Z was conserved as a complete covariate, and missingness was induced in X . Let $R_{X}$ indicate whether elements of X were missing ( $R_{X}=0$ ) or observed ( $R_{X}=1$ ). We varied the proportion of missing values as either ‘low’ with 10% missing, or ‘high’ with 50%. We defined four separate missingness mechanisms:

1. Missing completely at random (MCAR), defined as $P(R_{X}=0)=0.5$ or $P(R_{X}=0)=0.1$ .
2. Missing at random (MAR) conditional on Z , which was defined as $\mathrm{logit}\;P(R_{X}=0|Z)=\eta_{0}+\eta_{1}Z$ .
3. Outcome-dependent MAR (MAR-T), which was defined as $\mathrm{logit}\;P(R_{X}=0|T_{\text{stand}})=\eta_{0}+\eta_{1}T_{\text{stand}}$ . $T_{\text{stand}}$ is logT , standardised to have zero mean and unit variance. Note that T was the observed (event or censoring) time; if missingness depended on the true event time, this would lead to a missing not at random mechanism.
4. Missing not at random (MNAR) conditional on X , which was defined as $\mathrm{logit}\;P(R_{X}=0|X)=\eta_{0}+\eta_{1}X$ .

For mechanisms (2)–(4), $\eta_{1}$ represented the strength and direction of the missingness mechanism. For example, if $\eta_{1}<0$ in the MAR mechanism, observations with smaller values of the Z had a larger probability (increasing with more extreme $\eta_{1}$ ) of the corresponding X being missing. In the present study, we varied $\eta_{1}=\{-1,-2\}$ , representing ‘weak’ and ‘strong’ mechanisms, respectively. In this context, the MAR-T mechanism could reflect a measurement that is only collected if a subject survives long enough into a study and is in follow-up, as may be the case with a genetic test. Although this kind of measurement is collected or only available at a later point in time, it can still be considered as baseline information and does *not* constitute conditioning on the future.

The value of $\eta_{0}$ was chosen (in each simulated data set) such that the average missingness probability was equal to either 0.5 or 0.1. This was done via standard root-solving for a fixed value of $\eta_{1}$ .

#### 5.1.4 Design

The simulation study is chosen to follow a partially factorial design, where the parameters outlined above are varied systematically. A full factorial design would result in 4 (missingness mechanisms) × 2 (mechanism strengths) × 2 (proportions missing data) × 2 (covariate types for X ) × 2 (baseline hazard parametrisations) × 2 (effects magnitudes of X on cause-specific hazard of REL) =128 scenarios. However, the strength of the missingness mechanism cannot be varied for MCAR settings by definition, leaving 112 scenarios in total.

### 5.2 Estimands

The analysis models of interest are the cause-specific Cox proportional hazards models for REL and NRM, $h_{k}(t|X,Z)=h_{k0}(t)\exp\;(\beta_{k}X+\gamma_{k}Z)$ for k={1,2} . We then have two main sets of estimands of interest:

-
$\theta_{\text{regr}}=\{\beta_{1},\gamma_{1},\beta_{2},\gamma_{2}\}$ , which are the data-generating regression coefficients from both cause-specific Cox models.
-
$\theta_{\text{\textbackslash{},pred}}$ , which is a vector containing the REL and NRM probabilities (cumulative incidences) for a set of reference patients at 6 months, 5 years and 10 years after baseline.

These reference patients were defined by all combinations of $Z_{\text{ref}}=\{-1,0,1\}$ with $X_{\text{ref}}=\{-1,0,1\}$ for continuous X , and $X_{\text{ref}}=\{0,1\}$ for binary X . Since the data-generating coefficients for both competing events had a positive effect on the cause-specific hazards, one could for example refer to $\left\{X_{\text{ref}},Z_{\text{ref}}\}=\{1,1\right\}$ as a ‘high risk’ individual, and ‘low risk’ for {−1,−1} .

### 5.3 Methods

Five missing data methods were compared in each simulation scenario:

1.
CCA – an analysis run on a data set after listwise deletion.
2.
$CH_{1}$ – MI with imputation model predictors including Z , the event indicator solely for event one i.e. I(D=1) , and the cumulative hazard for REL ${\hat{H}}_{1}(T)$ (at the end of follow-up for each individual), based on the Nelson–Aalen estimator, as an approximation of the cumulative baseline hazard $H_{10}(T)$ .
3.
$CH_{12}$ – MI with imputation model predictors including Z , the event indicator D as a three level factor variable, and the cumulative hazards for both events ${\hat{H}}_{1}(T)$ and ${\hat{H}}_{2}(T)$ ; outlined in the ‘Fully conditional approach (MICE)’ section.
4.
$CH_{12,\text{Int}}$ – identical to the $CH_{12}$ , with the addition of the interactions ${\hat{H}}_{1}(T)\times Z$ and ${\hat{H}}_{2}(T)\times Z$ ; outlined in the Section 4.1.
5. SMC-FCS – the approach outlined in the Section 4.2, using Z as sole predictor in the X∣Z model (default setting).

The $CH_{1}$ method corresponds to the ‘FCS survival’ method explored in the simulation study by Bartlett and Taylor, where failures other than cause one are treated as censored and the cumulative hazard of cause two is omitted from the imputation model. It corresponds to a direct application of the White and Royston results^7^ to the cause-specific Cox model for cause one, which may present itself as intuitive when interest lies in a single failure cause.

Additionally, the model was also fitted on the complete data set prior to any missingness being induced in X . For the imputation methods, the number of imputed data sets was varied as m={5,10,25,50} . We set max(m)=50 since no substantial reduction in empirical standard errors was observed over trial runs with m=100 . We also note that for m≠50 , the imputations were not re-run independently. Results were instead pooled across the first 5, 10 or 25 imputed data sets from the original 50.

When X was continuous, the imputation model was linear regression. For binary X , the imputation model was logistic regression. We note that since there was only one partially observed covariate, chained equations were not needed. Nevertheless, we still refer to methods $CH_{1}$ , $CH_{12}$ and $CH_{12,\text{Int}}$ under the general umbrella term ‘MICE’ methods when reporting the results.

### 5.4 Performance measures

For $\theta_{\text{regr}}$ , we recorded the point estimates, empirical and estimated standard errors, absolute bias and coverage probabilities. As our primary measure of interest was bias, we based the number of simulation replications per scenario $n_{\text{sim}}$ on a desired Monte–Carlo standard error (MCSE) of bias. As per Morris et al.,^27^ this is defined as $\text{MCSE( Bias) }=\sqrt{\theta_{\text{regr}}/n_{sim}}$ . We assumed that $\text{SD}({\hat{\theta }}_{\text{regr}})\leq 0.125$ (largest empirical standard error to be expected with binary X , based on small trial run), and we deemed a MCSE( Bias) ≤0.01 to be appropriate. We thus required $n_{\text{sim}}=0.125^{2}/0.01^{2}\approx 156$ replications per scenario, which we rounded up to $n_{\text{sim}}=160$ . We thus generated 160 independent data sets per simulation scenario.

For ${\hat{\theta }}_{\text{\textbackslash{},pred}}$ , we recorded the point estimates, empirical standard errors, absolute bias, coverage probabilities and root mean square error (RMSE). We focus primarily on reporting bias and RMSE. Based on trial runs, we assumed $\text{SD}({\hat{\theta }}_{\text{\textbackslash{},pred}})\leq 0.05$ , which for 160 replications would result in a $\text{MCSE( Bias) }\leq 0.05/\sqrt{160}\approx 0.004$ . We thus proceeded with the same number of simulated data sets.

#### 5.4.1 Software

All analyses were performed using R version 3.6.2^28^. The substantive model compatible imputation was performed using the SMC-FCS package version 1.4.1,^29^ and MICE was performed using the mice package version 3.8.0.^30^ The cause-specific Cox models were run and subsequent predicted cumulative incidences were obtained using the mstate package version 0.2.12.^31^

### 5.5 Results

We focus primarily on $\beta_{1}$ (the regression coefficient for X in the cause-specific REL model) and the 5-year probabilities of REL and NRM. For the imputation methods, we present results only with m=50 . Full results are reported in the Supplemental materials, linked at the end of the present text.

#### 5.5.1 Regression coefficients

**Figure 1.**
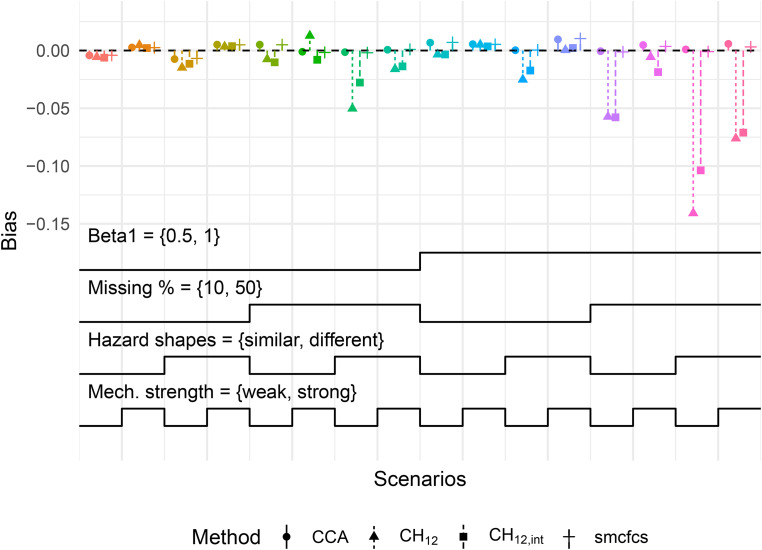
Bias in $\beta_{1}$ for MAR mechanism with continuous X . Each cluster of points corresponds to a scenario defined by the step functions at the bottom of the plot. Each step represents a level of a factor being varied and is read from left to right (e.g. for Hazard shapes, the first step is ‘similar’ while the second is ‘different’). Monte-Carlo standard errors of bias for all scenarios were below 0.008. Mech.: missingness mechanism; MIR: missing at random.

Figure 1 summarises the results with regard to bias in the estimation of $\beta_{1}$ with a MAR mechanism induced on continuous X . The plot is a variant of a nested-loop plot, where each colour-cluster of points represents a scenario defined by the step functions at the bottom of the plot.^32^ For example, the left-most bin in the plot corresponds to a scenario with data-generating $\beta_{1}=0.5$ , 10% missing data, similar hazard shapes and a weak missingness mechanism. For readability, the $CH_{1}$ method and the analysis ran on the full data set prior to inducing missing data are omitted from the Figure.

First, we note that in the 16 scenarios depicted, both CCA and SMC-FCS showed little to no bias in the estimation of $\beta_{1}$ . For CCA , no bias was expected given that this was a case of covariate-dependent MAR, and results for SMC-FCS were in line with the simulations of Bartlett and Taylor.^10^ Second, the MICE methods showed varying amounts of bias depending on the scenario. With increasing true covariate effects and a higher proportion of missing values, the bias was larger. This was to be expected in light of the approximations employed in the Section 4.1, which are valid for small covariate effects. Moreover, the magnitude of the bias was also larger when the baseline hazard shapes were different. Last, adding the interaction terms in the imputation model did not significantly reduce bias, except when the missingness mechanism was weak, and the baseline hazard shapes were different.

**Figure 2.**
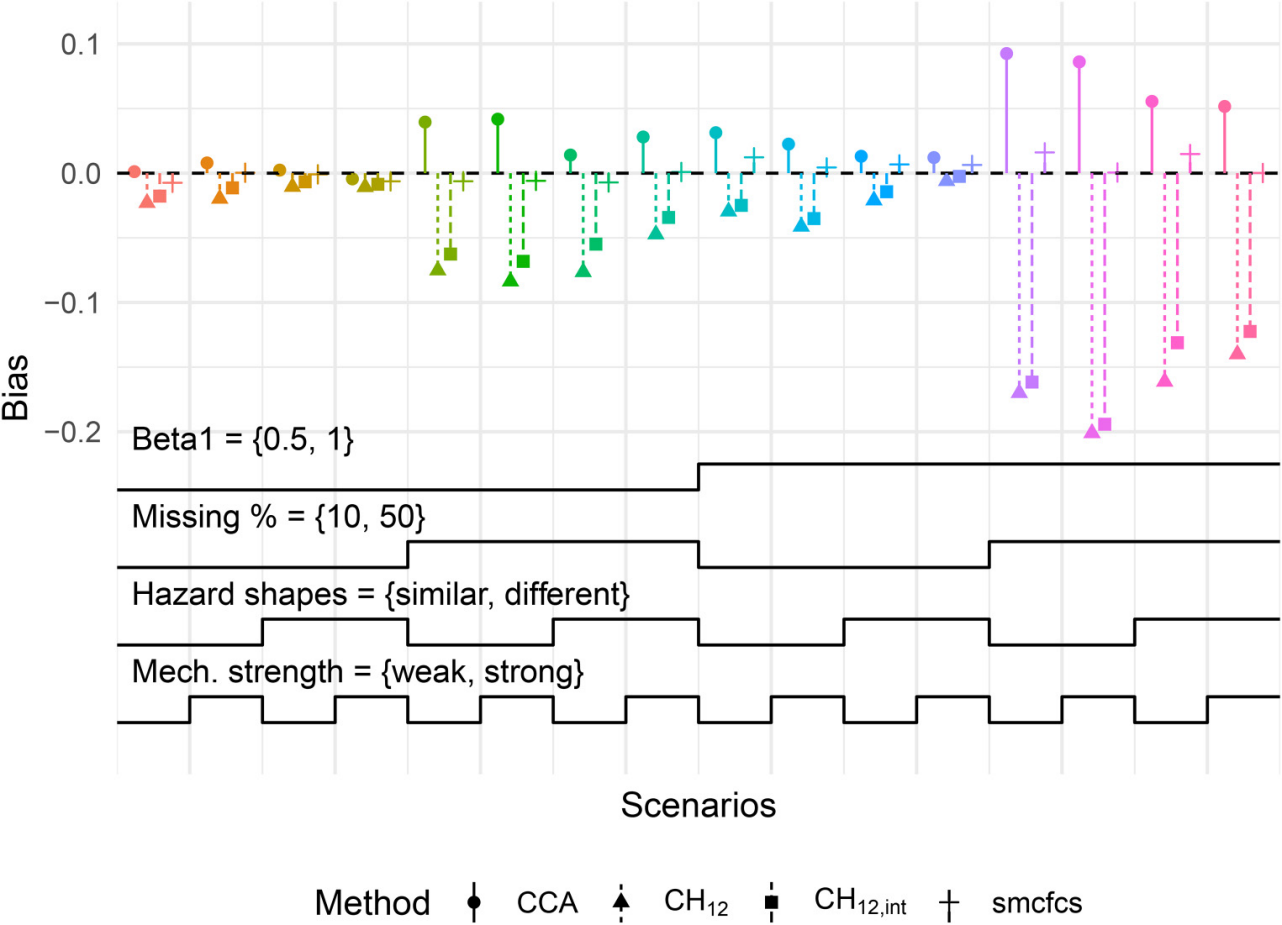
Bias in $\beta_{1}$ for MAR-T mechanism with continuous X . Monte-Carlo standard errors of bias for all scenarios were below 0.008. Refer to Figure 1 for a description on how to read this type of plot. Mech.: missingness mechanism; MAR-T: outcome-dependent missing at random.

In contrast, we also present the results for $\beta_{1}$ with a MAR-T mechanism in Figure 2, again with continuous X . In this case, CCA was consistently biased, as is expected when missingness is dependent on the outcome. Particularly for a high proportion of missing values, the bias in both MICE methods was even more severe than that of CCA , reaching close to 20% (relatively). Conversely, SMC-FCS was consistently unbiased across the depicted scenarios.

We also briefly summarise some of the more general findings across the simulations reported in the Supplemental material. First, efficiency gains (in the form of smaller estimated standard errors) were mainly observed for $\gamma_{1}$ and $\gamma_{2}$ . Second, the $CH_{1}$ method yielded the largest biases and lowest coverage probabilities of all methods. This was unsurprising, as $CH_{1}$ corresponded to imputing X as if competing outcomes were considered as censoring. Third, the findings with MCAR missingness were largely analogous to those of the MAR reported above; and in presence of MNAR, all imputation methods (including SMC-FCS) showed appreciable bias. Last, in scenarios with binary X , the overall bias in the MICE methods was lower with respect to scenarios with continuous X . This could be attributed to the different terms that are being approximated in the imputation models. In addition to the cumulative baseline hazards, only $\exp\;(\gamma_{k}Z)$ is being approximated in the case of binary X , whereas in the continuous case a fuller $\exp\;(\beta_{k}X+\gamma_{k}Z)$ is being approximated.

In terms of RMSE, which summarises both bias and variance, the differences in performance between the methods in M(C)AR scenarios wwere smaller, aside from when missingness was high and the baseline hazard shapes were different (see for example Figure 2.1.2 of the Supplemental material on regression coefficients).

#### 5.5.2 Predicted probabilities

**Figure 3.**
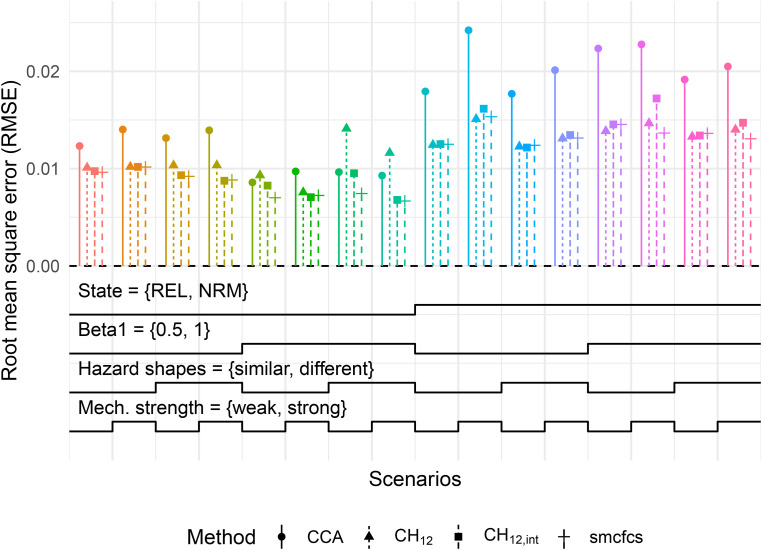
RMSE of 5-year REL and NRM probabilities with {X,Z}={−1,−1} for MAR with 50% missing values. Monte-Carlo standard errors of RMSE for all scenarios were below 0.002. Refer to Figure 1 for a description on how to read this type of plot. Mech.: missingness mechanism; RMSE: root mean square error; REL: relapse; MAR: missing at random; NRM: non-relapse mortality.

Concerning predicted probabilities, we focus on the estimation of 5-year REL and NRM probabilities for a ‘low-risk’ individual, i.e. {X,Z}={−1,−1} with continuous X . Figure 3 summarises the RMSE of these probabilities under a MAR mechanism where 50% of values are missing.

We point the reader to the y -axis of the plot, where results are now on the probability scale. The largest RMSE reported in the plot was just under 2.5%, with most RMSE values for the imputation methods being under 1.5%, with little to no difference between them. In these scenarios, the imputation methods outperform CCA , but with the finest of margins. This is part of a general finding across the simulations: the predicted probabilities when using the imputation methods overall had very little bias, and little reduction in variability was observed beyond m=25 imputations. We note that since all methods were similarly biased under M(C)AR (as seen for example in Figure 1.2.1 of the Supplemental material on predictions), the RMSE for CCA is expected to be a factor of $\sqrt{2}$ larger than for the imputation methods when missingness was ‘high’, given that CCA used half as much data.

We propose various explanations for this behaviour. First, we note that the prediction results for {X,Z}={0,0} (with X continuous or binary) can be taken as a proxy for how precisely the cause-specific baseline hazards are estimated. For all non-MNAR scenarios, little to no bias was found in the predicted probabilities for these reference patients. This may be additionally linked to the fact that X and Z are centred and normal, which could imply that $H_{k0}(T)$ is adequately approximated by the Nelson–Aalen estimator. Second, regarding regression coefficients, bias was primarily observed in $\beta_{1}$ and $\beta_{2}$ , with the former showing more extreme bias when data-generating $\beta_{1}=1$ . Estimates of $\gamma_{1}$ and $\gamma_{2}$ however generally only exhibited biases of up to 5% in the MAR scenarios, and slightly higher for CCA in MAR-T scenarios. Well-estimated cause-specific baseline hazards in tandem with close to unbiased estimates of $\gamma_{k}$ could then explain the small bias in the predictions, since bias in the linear predictor as a whole ( $\beta_{k}X+\gamma_{k}Z$ ) only reached 10% in the most extreme cases, and was mostly below the 5% mark otherwise.

#### 5.5.3 Additional simulations

In Supplemental material I (available online), we performed two additional simulation studies. The first investigated the use of the Breslow estimates of the cumulative baseline hazards in the imputation model, updated at each iteration of the imputation procedure. Consistent with earlier results in the standard survival setting, MICE using intra-iteration updates of the Breslow estimates performed no better than using the marginal cumulative hazards in the imputation model.^7^ The second study assessed the performance of the MI methods in the presence of K=3 competing events. In this setting, SMC-FCS remained unbiased, while the MICE methods including additional interaction terms performed slightly better than those without.

## 6 Illustrative analysis

We used the motivating alloHCT data set introduced in the Section 2 illustrate the methods described in the simulation study. Cause-specific Cox proportional hazards models were fitted for both REL and NRM, conditional on a set of baseline predictors chosen on the basis of substantive clinical knowledge. An overview of these predictors, including their names, descriptions and proportion of missing values, can be found in Appendix B. The same predictors were used in the models for REL and NRM.

We used the CCA , $CH_{12}$ and SMC-FCS methods to handle the missing baseline covariate data, which we assumed to be MAR. Given that $CH_{12,\text{Int}}$ did not show much improvement over $CH_{12}$ in the simulation study, we decided to use the more parsimonious latter. Therefore, the imputation model for a partially observed covariate using $CH_{12}$ contained as predictors the remaining fully and partially observed covariates from the substantive model, and the marginal cumulative hazards for both events. For SMC-FCS, the imputation model similarly contained the remaining fully and partially observed covariates from the substantive model, which is the default setting. Continuous covariates were imputed using linear regression, binary covariates using logistic regression, ordered categorical using proportional odds regression and nominal categorical using multinomial logistic regression. Since missingness spanned multiple covariates, chained equations were required.

To motivate the choice of m for $CH_{12}$ and SMC-FCS, we used von Hippel’s quadratic rule based on the fraction of missing information (FMI) rather than the proportion of complete cases.^33,34^ We first ran a set of m=20 imputations, with $n_{\text{iter}}=20$ iterations. After pooling, the coefficient with largest FMI was that of donor age in the model for NRM, with a value of approximately 0.49. Based on an 95% upper-bound for this FMI, and for a desired coefficient of variation of 0.05, we would require approximately m=84 imputed data sets. We rounded this upwards, and performed our final analysis with m=100 . We conserved $n_{\text{iter}}=20$ as convergence was generally observed from 10 iterations onwards.

**Figure 4.**
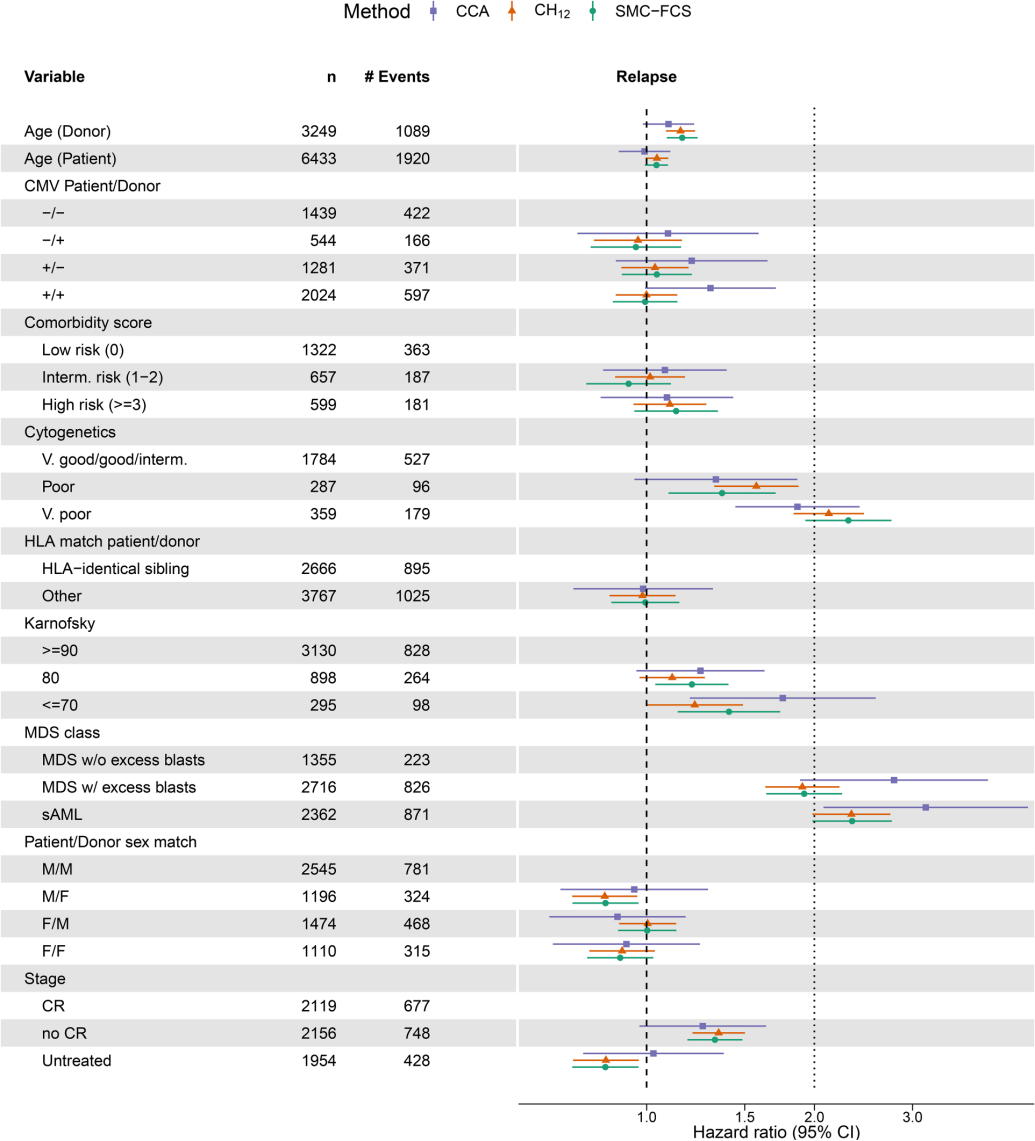
Forest plot with point estimates and 95% confidence interval for the cause-specific Cox model for Relapse. On the x -axis are the hazard ratios, which is plotted on the log scale where the confidence intervals are symmetric. Variables and their descriptions can be found in the data dictionary. Per level of factor and for continuous variables, we show the observed counts ( n ) and the number of relapse events (# Events) in the full data set.

**Figure 5.**
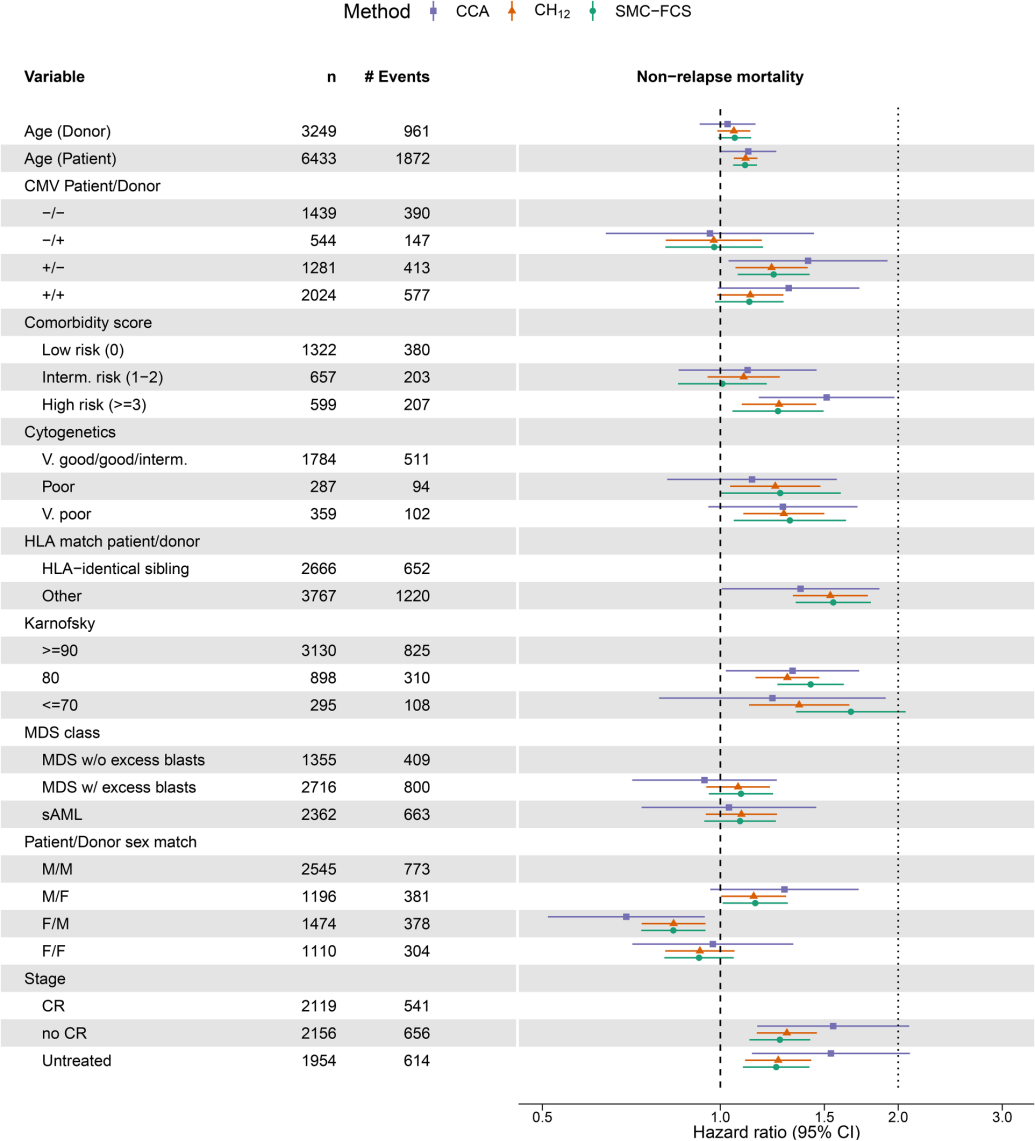
Forest plot with point estimates and 95% confidence interval for the cause-specific Cox model for non-relapse mortality (NRM). On the x -axis are the hazard ratios, which is plotted on the log scale where the confidence intervals are symmetric. Variables and their descriptions can be found in the data dictionary. Per level of factor and for continuous variables, we show the observed counts ( n ) and the number of NRM events (# Events) in the full dataset.

Figure 4 summarises the exponentiated point estimates (hazard ratios, HR) and associated 95% confidence intervals (CI) from the cause-specific model for REL. The CIs for $CH_{12}$ and SMC-FCS are based on the pooled standard errors and the t -distribution. First, we observed a clear gain in efficiency across all coefficients for both imputation methods relative to CCA . Second, there was general agreement between the estimates obtained from both $CH_{12}$ and SMC-FCS; a finding which was also reported in the illustrative analysis in the work by Bartlett and Taylor.^10^ Third, we did note some differences between CCA and the imputation methods for certain variables, such as remission status or Karnofsky score. The most surprising case of this was with the MDS class of the patient, which was completely observed. In the model for REL, the HR for the sAML category estimated with CCA is just above three, whereas the imputation methods estimate it much closer to two. This also raises the point that for categorical variables, differences in methods can be seen on the category level rather than on the variable level as a whole – as also evidenced by the estimated HRs for the cytogenetics variable. Results for the cause-specific NRM model are summarised in Figure 5.

Furthermore, we computed the predicted 5-year cumulative incidences of REL and NRM for a set of three reference patients. These corresponded to the three MDS classes, all with the median patient and donor ages at transplant, and with reference levels for the remaining categorical covariates. Table 1 summarises the point estimates, and corresponding 95% CIs. For the imputation methods, the variances of the predicted probabilities were obtained with the Aalen estimator.^35^ Subsequently, the 95% CIs were constructed after transformation on the complementary log-log scale, as described in the work of Morisot and colleagues.^36^ For comparability, the CIs for CCA were also constructed on the complementary log–log scale. In line with the results from the estimated regression coefficients, both imputation methods yielded quasi identical results. By contrast, CCA yielded cumulative incidences that were generally lower by approximately 3–7 percentage points, with CIs that were up to twice as wide.

**Table 1.** Predicted cumulative incidence (%) of both REL and NRM at 5-years for three reference patients with different MDS classes, reference levels for categorical covariates and sample median values for continuous covariates. The 95% confidence intervals were constructed based on a complementary log-log transformation.

MDS class	CC	$CH_{12}$	SMC-FCS
**REL**			
MDS without excess blasts	10.7 [6.5; 17.2]	17.2 [14.1; 20.8]	17.1 [13.9; 20.9]
MDS with excess blasts	26.9 [19.4; 36.4]	29.7 [25.8; 34.2]	29.7 [25.6; 34.4]
sAML	29.7 [21.4; 40.2]	34.9 [30.7; 39.6]	34.9 [30.4; 39.8]
**NRM**			
MDS without excess blasts	15.1 [9.7; 23]	18.1 [15.1; 21.7]	17.8 [14.7; 21.5]
MDS with excess blasts	13.1 [9; 18.8]	17.8 [15.2; 20.8]	17.7 [14.9; 20.9]
sAML	14.0 [9.5; 20.4]	17.4 [14.9; 20.2]	17.0 [14.4; 20]

REL: relapse; NRM: non-relapse mortality; MDS: myelodysplastic syndromes; sAML: secondary acute myeloid leukemia.

Such differences between the MI methods and CCA do question the validity of the M(C)AR assumption made. In the EBMT registry, many missing values can be considered MCAR, for reasons relating to data management. Variables such as comorbidity score, cytogenetic classification and donor age became more frequently collected over time as their clinical relevance grew clearer. Missingness may also be related to the transplant centre, i.e. particular measurements not being recorded in certain clinics. In the current analysis, both calendar date and transplant centre (categorical, large number of levels) were not included in the imputation model for simplicity. An option would have been to include them as auxiliary variables (added as predictor to X∣Z , but not to substantive model), however, the use of auxiliary variables was not a focus of this manuscript, and both MICE and SMC-FCS make different assumptions with respect to the inclusion of these variables in the imputation model. Specifically, SMC-FCS would assume independence of centre and outcome given the covariates in the substantive model – an assumption which likely does not hold in the registry.^37^

## 7 Discussion

In this paper, we assessed the performance of currently implemented MI methods, MICE and SMC-FCS, that deal with missing baseline covariate data when the analysis model of interest is a cause-specific Cox proportional hazards model. For the MICE approach, we provided motivation for the imputation models to be used for continuous, binary, multi-level nominal and ordered categorical covariates with missing values. This is an extension of the work of White and Royston on Cox proportional hazards models for standard survival outcomes.^7^

We covered a wide range of scenarios in our simulation study, also investigating parameters commonly not addressed in simulation studies for this or similar problems, such as the shape of the baseline hazard and strength of association in the missingness model. Our results confirm the findings of the earlier work of Bartlett and Taylor.^10^ Namely, in terms of estimating regression coefficients, SMC-FCS categorically outperforms MICE across all investigated non-MNAR scenarios. Adding the ${\hat{H}}_{k}(T)\times Z$ interactions in the imputation model improves performance somewhat, but the substantial bias remains. When using MICE, bias grows more extreme as both covariate effects and the proportion of missing values increase and seems also affected by the shape of the baseline hazards and the strength of the missingness mechanism. Interestingly, in scenarios where missingness was outcome-dependent, the MICE approach produced biases even larger than those with CCA, which is expected to be biased in these scenarios. Although this is clearly concerning, we do acknowledge that given the longitudinal nature of survival data, a missingness mechanism that depends on the observed event time may be rare.

To the best of our knowledge, our work is the first systematic assessment of the performance of MI for missing covariates with regard to the prediction of cumulative incidences. In this respect, the imputation methods performed comparably, which may be attributed to a solid estimation of both the baseline hazards and of the regression coefficients from the complete covariates. The low biases found are consistent with those reported in the work by Mertens et al.^22^ on MI and prediction in the context of logistic regression. Furthermore, empirical standard errors did not become smaller beyond around m=50 imputed data sets. If interest lies in reducing the variability of individual predictions between replications of an MI procedure, or replications of a particular study, a choice of m in the order of hundreds will likely be required, as suggested by the same work by Mertens and colleagues. We also emphasise that since we are predicting for reference patients (for which we have *true* data-generating probabilities over time), the assessment of the estimated probabilities is not hindered by any optimism that we would need to correct for, using for example a cross-validation procedure.

There are various limitations to the present work. First, we remark that the explored scenarios are naturally limited as a result of the vast possible parameter space for simulation studies in the field of missing data. For example, missingness was only induced in a single variable. Naturally, more realistic data will be subject to missingness across multiple variables, among which could be interactions in the substantive model. Second, the imputation of covariates with more complex distributions (conditional on other variables) fell outside of the scope of this work. There is a clear need for research and guidance on how to properly impute such variables, particularly for continuous measurements which are heavily skewed.^38^ This may in turn prevent unnecessary categorisation of these variables, and thus further loss of power. Last, we note that in the illustrative analysis, various multi-level nominal and ordinal categorical variables were multiply imputed. These covariate types were not investigated in the simulation study, but are pertinent for further research. Avenues for further exploration could include issues like category inbalance, and comparisons between imputing with proportional odds, multinomial logistic and even a latent normal model.^39,40^

Furthermore, a noteworthy difference between the MICE and SMC-FCS approaches in the present context lies in the treatment of cumulative cause-specific baseline hazards functions $H_{k0}(T)$ . While the SMC-FCS approach updates $H_{k0}(T)$ at each iteration of the imputation procedure using the Breslow estimate, the MICE approach approximates $H_{k0}(T)$ once using the Nelson–Aalen estimate and keeps them fixed throughout the imputation procedure. Updating $H_{k0}(T)$ iteratively with MICE was investigated in the single event setting by White and Royston, with simulations failing to justify its use over the inclusion of the Nelson–Aalen estimates in the imputation model.^7^ The additional simulation study reported in Supplemental material I of the present work appears to show that these earlier results do extend to the competing risk setting. This in turn suggests that the differences in performance between MICE and SMC-FCS could almost entirely be attributed to the functional form of the imputation model, rather than to any error in estimating $H_{k0}(T)$ .

For practising statisticians, our work in combination with that of Bartlett and Taylor^10^ shows that SMC-FCS should be the current standard when applying MI in the cause-specific competing risks setting. Although in many controlled situations differences between MICE and SMC-FCS may be small (as in our alloHCT example), the latter seems to be the safest choice given the inherent lack of knowledge regarding the true underlying missingness mechanism. Naturally, SMC-FCS can still be biased, and so the researcher is encouraged to think meticulously about the assumptions underlying their data. We also recommend that a CCA still be a starting point before performing MI, as it will be unbiased when M(C)AR and covariate-dependent MNAR hold. When biases occur, they may not be as extreme as expected, particularly when the proportion of incomplete cases is low. However, in applications where the proportion of incomplete cases is very high and the M(C)AR assumption is deemed plausible, efficiency gains can be substantial when using MI. This was particularly the case in our alloHCT example, where smaller standard errors were observed with the MI methods for both regression coefficients and predicted cumulative incidence.

The present findings add to a broader literature concerning missing covariates in the context of Cox models.^41–43^ Studies investigating methods for dealing with missing covariates for a substantive Fine-Gray model remain scarce. For the Fine-Gray model, MI has predominantly been assessed in the context of missing or interval-censored outcomes.^44,45^ We conclude by remarking that likelihood-based and fully Bayesian approaches have also not yet been explored or implemented in the context of competing risks, despite already showing promise in other applications.^46^

## Supplemental Material

sj-pdf-1-smm-10.1177_09622802221102623 - Supplemental material for Multiple imputation for cause-specific Cox models: Assessing methods for estimation and prediction Click here for additional data file. Supplemental material, sj-pdf-1-smm-10.1177_09622802221102623 for Multiple imputation for cause-specific Cox models: Assessing methods for estimation and prediction by Edouard F Bonneville, Matthieu Resche-Rigon, Johannes Schetelig, Hein Putter and Liesbeth C de Wreede in Statistical Methods in Medical Research

## Appendix A

### Imputation model derivations

Without loss of generality, we assume that X and Z are scalars. The following derivations are valid under the MAR assumption, but also apply if the missing data are MCAR. Letting $R_{X}$ indicate whether elements of X are missing ( $R_{X}=0$ ) or observed ( $R_{X}=1$ ), MAR implies $R_{X}⊥⊥X|\{T,D,Z\}$ while for MCAR $R_{X}⊥⊥X$ .

We can express the log conditional density of the (right-censored) competing risks outcomes given the covariate data as

$$\log\;p(T,D|X,Z)=\log\;S(T|X,Z)+\sum_{k=1}^{K}I(D=k)\log\;h_{k}(T|X,Z).$$

Using $\log\;S(T|X,Z)=-\sum_{k=1}^{K}H_{k}(T|X,Z)$ , and assuming a cause-specific Cox proportional hazards model for each failure cause k as $h_{k}(t|X,Z)=h_{k0}(t)\exp\;(\beta_{k}X+\gamma_{k}Z)$ , we can write

$$\log\;p(T,D|X,Z)=\sum_{k=1}^{K}\{I(D=k)[\log\;h_{k0}(T)+(\beta_{k}X+\gamma_{k}Z)]-H_{k0}(T)\exp\;(\beta_{k}X+\gamma_{k}Z)\}.$$

We can then plug-in the above expression into Equation (3) describing the fully conditional density of X , yielding

$$\log\;p(X|T,D,Z)=\log\;p(X|Z)+\sum_{k=1}^{K}I(D=k)(\beta_{k}X+\gamma_{k}Z)-\sum_{k=1}^{K}H_{k0}(T)\exp\;(\beta_{k}X+\gamma_{k}Z)+c,$$

where c may depend on T , D or Z , but not on X . We note that if Z is a categorical variable with more than two levels, $\gamma_{k}$ represents a vector of coefficients for the dummy codes of Z .

#### Binary X

Suppose X is a binary covariate, depending on Z through a logistic regression model $\mathrm{logitP}(X=1|Z)=\zeta_{0}+\zeta_{1}Z$ . Given this missing data model, the objective now is to derive an expression for logitP(X=1∣T,D,Z) . In general we have that

(8)
$$\begin{array}{rl} \mathrm{logitP}(X=1|T,D,Z) & =\log\;p(T,D|X=1,Z)-\log\;p(T,D|X=0,Z)+\mathrm{logitP}(X=1|Z)\\ & =\zeta_{0}+\zeta_{1}Z+\sum_{k=1}^{K}I(D=k)\beta_{k}-\sum_{k=1}^{K}H_{k0}(T)\exp\;(\gamma_{k}Z)(e^{\beta_{k}}-1). \end{array}$$

If Z is categorical with J≥2 levels (0,…,J−1) , the above expression extends to

$$\begin{array}{rl} \mathrm{logitP}(X=1|T,D,Z) & =\alpha_{0}+\sum_{k=1}^{K}\alpha_{k}I(D=k)+\sum_{k=1}^{K}\alpha_{K+k}H_{k0}(T)\\ & \;+\sum_{\;j=1}^{J-1}\alpha_{2K+j}I(Z=j)+\sum_{\;j=1}^{J-1}\sum_{k=1}^{K}\alpha_{(j+1)K+(J-1)+k}I(Z=j)H_{k0}(T), \end{array}$$

which suggests that for categorical Z we can impute missing X values using a logistic regression with D (as a factor variable), the cumulative baseline hazards, evaluated at T , for all causes of failure as covariates, Z (as a factor variable), and the complete interactions between the cumulative baseline hazards at T and Z . The number of parameters, including intercept, equals JK+J+K . For example, if there are K=2 competing risks and Z has J=3 categories, there are 11 logistic regression parameters to be estimated (intercept included).

If Z is continuous, there are no exact results due to the $\exp\;(\gamma_{k}Z)$ terms. Analogously to White and Royston, we use a first order Taylor series around $\bar{Z}$ to approximate it as $\exp\;(\gamma_{k}Z)\approx \exp\;(\gamma_{k}\bar{Z})[1+\gamma_{k}(Z-\bar{Z})]$ , which is valid if $\text{Var}(\gamma_{k}Z)$ is small.^7^ We can thus write

$$\begin{array}{rl} \mathrm{logitP}(X=1|T,D,Z) & \approx \zeta_{0}+\zeta_{1}Z+\sum_{k=1}^{K}I(D=k)\beta_{k}-\sum_{k=1}^{K}H_{k0}(T)(e^{\beta_{k}}-1)\exp\;(\gamma_{k}\bar{Z})(1-\gamma_{k}\bar{Z})\\ & \;-\sum_{k=1}^{K}H_{k0}(T)Z\gamma_{k}(e^{\beta_{k}}-1)\exp\;(\gamma_{k}\bar{Z})\\ & =\alpha_{0}+\sum_{k=1}^{K}\alpha_{k}I(D=k)+\sum_{k=1}^{K}\alpha_{K+k}H_{k0}(T)+\sum_{k=1}^{K}\alpha_{2K+k}H_{k0}(T)Z\\ & \;+\alpha_{3K+1}Z. \end{array}$$

This suggests an approximate imputation model for binary X and continuous Z including the following predictors: Z , D (as a factor) and all $H_{k0}(T)$ . Adding an interaction between all $H_{k0}(T)$ and Z improves the accuracy of this approximation.

#### Nominal categorical X

Suppose X is categorical with J>2 levels. If we assume the variable to be unordered, we can specify a polytomous logistic regression (also known as multinomial regression) for p(X∣Z) . The model can be expressed in log odds form as

$$\log\;\frac{P(X=j|Z)}{P(X=0|Z)}=\zeta_{\;j0}+\zeta_{\;j1}Z.$$

By coding the reference category as X=0 , analogously to (8) we obtain

$$\log\;\frac{P(X=j|T,D,Z)}{P(X=0|T,D,Z)}=\zeta_{\;j0}+\zeta_{\;j1}Z+\sum_{k=1}^{K}I(D=k)\beta_{\;jk}-\sum_{k=1}^{K}H_{k0}(T)\exp\;(\gamma_{k}Z)(e^{\beta_{\;jk}}-1).$$

From this it becomes clear that all derivations and approximations of logitP(X=1∣T,D,Z) for binary X continue to hold for $\log\;\frac{P(X=j|T,D,Z)}{P(X=0|T,D,Z)}$ , replacing $\zeta_{0}$ and $\zeta_{1}$ by $\zeta_{\;j0}$ and $\zeta_{\;j1}$ , and $\alpha_{k}$ ’s by $\alpha_{\;jk}$ ’s. This implies that the imputation model for an unordered categorical X should contain the same predictors as for a binary X . The above expression is again exact, given no Z , and also for categorical Z , provided the full interactions between the levels of Z and the cumulative baseline hazards are used; the expression for categorical Z with more than 2 levels extends in the same way.

#### Ordered categorical X

We consider X as categorical with J>2 ordered levels. A proportional odds model can then be specified for p(X∣Z) , which can be expressed by

$$\log\;\frac{P(X\leq j|Z)}{1-P(X\leq j|Z)}=\zeta_{\;j}+\zeta_{Z}Z,$$

or simply $\mathrm{logitP}(X\leq j|Z)=\zeta_{\;j}+\zeta_{Z}Z$ for j={1,…,J−1} .

To motivate the fully conditional imputation model for X , we need an expression for logitP(X≤j∣T,D,Z) . This involves specifying p(T,D∣X≤j,Z) , which no longer has a proportional hazards density, but instead a *weighted sum* of proportional hazards densities. The imputation model for an ordered categorical X thus does not have a simple extension from the binary case. Nonetheless, including the cumulative cause-specific baseline hazards, D as a factor variable and the remaining covariates in the imputation model is still reasonable as an ad hoc solution.

#### Continuous X

In the case of a continuous X , we specify an exposure model $X|Z∼N(\zeta_{0}+\zeta_{1}Z,\sigma^{2})$ . We can write

(9)
$$\begin{array}{rl} \log\;p(X|T,D,Z) & =\sum_{k=1}^{K}I(D=k)\beta_{k}X-\sum_{k=1}^{K}H_{k0}(T)\exp\;(\beta_{k}X+\gamma_{k}Z)\\ & \;-\frac{X^{2}-2X(\zeta_{0}+\zeta_{1}Z)}{2\sigma^{2}}+c, \end{array}$$

where the terms that do not depend on X from the normal density are subsumed into c . Note that in what follows, the constant c is used to present anything that is not a function of X , and as such may not be equal from line to line. With the same reasoning as in the previous section, a bivariate Taylor approximation is used for the $\exp\;(\beta_{k}X+\gamma_{k}Z)$ around the sample means $\bar{X}$ and $\bar{Z}$ . By taking $y=\beta_{k}(X-\bar{X})+\gamma_{k}(Z-\bar{Z})$ , we can write the quadratic approximation as

$$\exp\;(\beta_{k}X+\gamma_{k}Z)\approx \exp\;(\beta_{k}\bar{X}+\gamma_{k}\bar{Z})[1+y+\frac{1}{2}y^{2}].$$

Using first the linear component of the above approximation in Equation (9) yields

(10)
$$\begin{array}{rl} \log\;p(X|T,D,Z) & \approx \sum_{k=1}^{K}I(D=k)\beta_{k}X-\sum_{k=1}^{K}H_{k0}(T)\beta_{k}X\exp\;(\beta_{k}\bar{X}+\gamma_{k}\bar{Z})\\ & \;-\frac{X^{2}-2X\zeta_{0}-2X\zeta_{1}Z}{2\sigma^{2}}+c,\\ & =\frac{2\sigma^{2}\sum_{k=1}^{K}I(D=k)\beta_{k}X-2\sigma^{2}\sum_{k=1}^{K}H_{k0}(T)\beta_{k}X\exp\;(\beta_{k}\bar{X}+\gamma_{k}\bar{Z})}{2\sigma^{2}}\\ & \;-\frac{X^{2}-2X\zeta_{0}-2X\zeta_{1}Z}{2\sigma^{2}}+c, \end{array}$$

and hence

$$\begin{array}{rl} & \log\;p(X|T,D,Z)\approx \\ & \;-\frac{X^{2}-2X[\overset{\alpha_{0}}{\overbrace{\zeta_{0}}}+\overset{\alpha_{1}}{\overbrace{\zeta_{1}}}Z+\sum_{k=1}^{K}I(D=k)\overset{\alpha_{k+1}}{\overbrace{\beta_{k}\sigma^{2}}}+\sum_{k=1}^{K}H_{k0}(T)\overset{\alpha_{K+k+1}}{\overbrace{\left(-\beta_{k})\sigma^{2}\exp\;(\beta_{k}\bar{X}+\gamma_{k}\bar{Z}\right)}}]}{2\sigma^{2}}\\ & \;+c. \end{array}$$

Thus, approximately

$$X|T,D,Z∼N(\alpha_{0}+\alpha_{1}Z+\sum_{k=1}^{K}\alpha_{k+1}I(D=k)+\sum_{k=1}^{K}\alpha_{K+k+1}H_{k0}(T),\sigma^{2}).$$

Based on a linear approximation for $\exp\;(\beta_{k}X+\gamma_{k}Z)$ , the suggested imputation model for missing X is a linear regression containing Z , D (as a factor variable) and all $H_{k0}(T)$ as covariates. This approximation is valid for small $\mathrm{Var}(\beta_{k}X+\gamma_{k}Z)$ .

For a more precise imputation model, we can revisit (10) and add the remaining terms from the quadratic part of the approximation (that do not depend on X ). After setting $w=\exp\;(\beta_{k}\bar{X}+\gamma_{k}\bar{Z})$ , adding the quadratic terms yields

$$\begin{array}{rl} \log\;p(X|T,D,Z) & \approx \sum_{k=1}^{K}I(D=k)\beta_{k}X-\frac{X^{2}-2X\zeta_{0}-2X\zeta_{1}Z}{2\sigma^{2}}\\ & \;-\sum_{k=1}^{K}H_{k0}(T)w[\beta_{k}X+\frac{1}{2}\beta_{k}^{2}(X-\bar{X}{)}^{2}+\beta_{k}\gamma_{k}X(Z-\bar{Z})]+c. \end{array}$$

After adjusting by $2\sigma^{2}$ and adding $-\sum_{k=1}^{K}H_{k0}(T)w(\beta_{k}^{2}{\bar{X}}^{2}/2)$ to c , we can write

$$\begin{array}{rl} \log\;p(X|T,D,Z) & \approx -\frac{X^{2}[1+\sigma^{2}\sum_{k=1}^{K}\beta_{k}^{2}H_{k0}(T)]}{2\sigma^{2}}\\ & \;+2X\times \{\frac{\zeta_{0}+\zeta_{1}Z+\sigma^{2}\sum_{k=1}^{K}\beta_{k}[I(D=k)-H_{k0}(T)w(1-\beta_{k}\bar{X})]}{2\sigma^{2}}\}\\ & \;-2X\times \{\frac{\sigma^{2}\sum_{k=1}^{K}H_{k0}(T)w\beta_{k}\gamma_{k}(Z-\bar{Z})}{2\sigma^{2}}\}+c. \end{array}$$

Thus, the conditional distribution of X , given (T,D,Z) is approximately normal with mean

$$\frac{\zeta_{0}+\zeta_{1}Z+\sigma^{2}\sum_{k=1}^{K}\beta_{k}[I(D=k)-H_{k0}(T)w(1-\beta_{k}\bar{X})]-\sigma^{2}\sum_{k=1}^{K}H_{k0}(T)w\beta_{k}\gamma_{k}(Z-\bar{Z})}{1+\sigma^{2}\sum_{k=1}^{K}\beta_{k}^{2}H_{k0}(T)},$$

and variance

$$\frac{\sigma^{2}}{1+\sigma^{2}\sum_{k=1}^{K}\beta_{k}^{2}H_{k0}(T)}.$$

Based on a quadratic approximation for $\exp\;(\beta_{k}X+\gamma_{k}Z)$ , the suggested imputation model for missing X is a linear regression containing Z , D (as a factor variable), all $H_{k0}(T)$ and all $H_{k0}(T)Z$ interactions as covariates. As explained by White and Royston, this is only valid by ignoring terms in $\beta_{k}^{2}$ and for small $\sigma^{2}\sum_{k=1}^{K}\beta_{k}^{2}H_{k0}(T)$ .^7^ We also note that the variance of X∣T,D,Z is non-constant in time.

### Appendix B Data dictionary

See Table 2.

**Table 2.** Data dictionary with predictor variables and their descriptions, levels and proportion missing data.

Variable	Description	Levels	% Missing	Summary
Age (Donor)	Donor age at alloHCT (decades)		49.49	4.21 (3.12, 5.25)
Age (Patient)	Patient age at alloHCT (decades)		0	5.6 (4.69, 6.19)
CMV Patient/Donor	CMV status in patient and donor	−/−		1439 (27%)
		−/+		544 (10%)
		+/−		1281 (24%)
		+/+	17.8	2024 (38%)
Comorbidity score	HCT-CI score	Low risk (0)		1322 (51%)
		Interm. risk (1-2)		657 (25%)
		High risk ( ≥ 3)	59.93	599 (23%)
Cytogenetics	Cytogenetics categories used for IPSS-R	V. good/good/interm.		1784 (73%)
		Poor		287 (12%)
		V. poor	62.23	359 (15%)
HLA match patient/donor	HLA match between patient and donor	HLA-identical sibling		2666 (41%)
		Other	0	3767 (59%)
Karnofsky	Karnofsky performance status	≥ 90		3130 (72%)
		80		898 (21%)
		≤ 70	32.8	295 (7%)
MDS class	MDS groups based on subclassification at alloHCT	MDS w/o excess blasts		1355 (21%)
		MDS w/ excess blasts		2716 (42%)
		sAML	0	2362 (37%)
Patient/Donor sex match	Sex match patient and donor	M/M		2545 (40%)
		M/F		1196 (19%)
		F/M		1474 (23%)
		F/F	1.68	1110 (18%)
Stage	Stage at alloHCT	CR		2119 (34%)
		no CR		2156 (35%)
		Untreated	3.17	1954 (31%)

The ‘Summary’ column reports median and interquartile range for continuous variables, as well as counts and proportion per level of categorical variables. CMV: cytomegalovirus; CR: complete remission; IPSS-R: International Prognostic Scoring System; V.: very; interm.: intermediate; HLA: human leukocyte antigen; HCT-CI: hematopoietic stemcell transplantation-comorbidity index; M: male; F: female; MDS: myelodysplastic syndromes; sAML: secondary acute myeloid leukemia; w/: with; w/o: without.

### Non-relapse mortality – forest plot

See Figure 5.
