# Supplementary material for: Multiple imputation for cause-specific Cox models: Assessing methods for estimation and prediction
Source: Stat Methods Med Res. 2022 Jun 5;31(10):1860–80. doi: 10.1177/09622802221102623 (PMC9523822; doi:10.1177/09622802221102623)
Supplement: sj-pdf-1-smm-10.1177_09622802221102623 - Supplemental material for Multiple imputation for cause-specific Cox models: Assessing methods for estimation and prediction [file sj-pdf-1-smm-10.1177_09622802221102623.pdf]

Supplementary material I

Edouard F. Bonneville et al.

The present document contain the results of two additional simulation studies, while supplementary material II contains results from all simulations from the main manuscript, and is hosted using Github here: `https://github.com/survival-lumc/CauseSpecCovarMI`. Any figures from supplementary material II referenced in the manuscript are also included in the present supplementary file.

1 Supplementary simulation studies

We set up two additional simulation studies, to investigate a) the added value of intra-iteration updates of the cause-specific cumulative baseline hazards using the Breslow estimator, and b) how all described imputation methods perform in the presence of more than two competing events. The following sixteen scenarios are investigated in both studies:

β_1	Level X	Missing mech.	Mech. strength
0.5	Continuous	MAR	Weak
1.0	Continuous	MAR	Weak
0.5	Binary	MAR	Weak
1.0	Binary	MAR	Weak
0.5	Continuous	MAR-T	Weak
1.0	Continuous	MAR-T	Weak
0.5	Binary	MAR-T	Weak
1.0	Binary	MAR-T	Weak
0.5	Continuous	MAR	Strong
1.0	Continuous	MAR	Strong
0.5	Binary	MAR	Strong
1.0	Binary	MAR	Strong
0.5	Continuous	MAR-T	Strong
1.0	Continuous	MAR-T	Strong
0.5	Binary	MAR-T	Strong
1.0	Binary	MAR-T	Strong

Table 1: Scenarios overview for supplementary simulations. β_1 in this context still corresponds to the covariate effect of X in the cause-specific model of the first cause. Mech. = mechanism.

Kept fixed in both studies are the proportion of missing values in X (50%), the sample size of $n = 2000$, the number of multiple imputations $m = 50$, and the use of the 'different' hazards parametrization for causes 1 and 2. The complete covariate Z also remains continuous, and regression coefficients aside from β_1 are fixed as per the main manuscript. The primary

estimand is the bias in the estimated regression coefficients. The specific set-up for both studies are detailed in the next subsections.

1.1 Iteratively re-estimated Breslow

The MICE imputation methods described in the manuscript all require a working estimate of each $H_{k0}(T)$, the cause-specific cumulative baseline hazards. Thus far, we approximated these using the marginal cumulative hazards obtained with the Nelson-Aalen estimator. Instead, we can use the estimated cumulative baseline hazards obtained with the Breslow estimator, and include them in the imputation model. Furthermore, these can be updated at each iteration of the imputation procedure, conditioning on the latest imputations of X. Without updating (or using too few iterations), the cumulative baseline hazard estimates may be unsatisfactory.

White and Royston investigated this iterative Breslow approach in the standard survival setting, where it performed no better (and sometimes worse) than the Nelson-Aalen approaches [1]. In this present study, we investigate whether this is also the case for cause-specific Cox models. Using the scenarios outlined in Table 1, we compare the following methods:

1. CC - an analysis run on a dataset after listwise deletion.

2. CH_{12} - MI with imputation model predictors including Z, the event indicator D as a three level factor variable, and the cumulative hazards for both events $\hat{H}_1(T)$ and $\hat{H}_2(T)$.

3. $CH_{12,\text{Bres}}$ - identical to CH_{12}, except that instead of the marginal cumulative hazards, the Breslow estimate of each $\hat{H}_{k0}(T)$ (the cumulative cause-specific baseline hazards), are included in the imputation model. These are re-estimated at each iteration of the imputation procedure, set here to $n_{\text{iter}} = 10$.

4. $CH_{12,\text{True}}$ - identical to $CH_{12,\text{Bres}}$, except the *true* cumulative baseline hazards (obtained by integrating the known Weibull hazard) $H_{k0}(T)$ are used in the imputation model. While this is not an approach that can be used in practice, it eliminates any possible error in estimating $H_{k0}(T)$, and any remaining bias in the estimated regression coefficients would have to be attributed to the approximations made in the imputation model. This would practically reflect the cost of approximate compatibility between the analysis and imputation model.

5. smcfcs - the approach outlined in the manuscript, using Z as sole predictor in the $X \mid Z$ model (default setting).

The results are summarized in Figure 1. All three MICE methods CH_{12}, $CH_{12,\text{True}}$ and $CH_{12,\text{Bres}}$ performed essentially equally across all scenarios, and for all regression coefficients. The results are clearly consistent with the finding in the White and Royston article: the extra computation time needed for the intra-iteration updating of the cumulative baseline hazards is unjustified.

Briefly, it is worth discussing why method $CH_{12,\text{True}}$ did not outperform CH_{12} at all. If we are describing the estimated marginal cumulative hazards as approximations of the cumulative baseline hazards, we could conjecture that $CH_{12,\text{True}}$ should always perform better or at least as well as CH_{12} - particularly in situations with large cumulative incidences and strong covariate effects. To understand why this may not the case, consider the case with binary X and continuous Z. Recall Equation (8) from the manuscript as

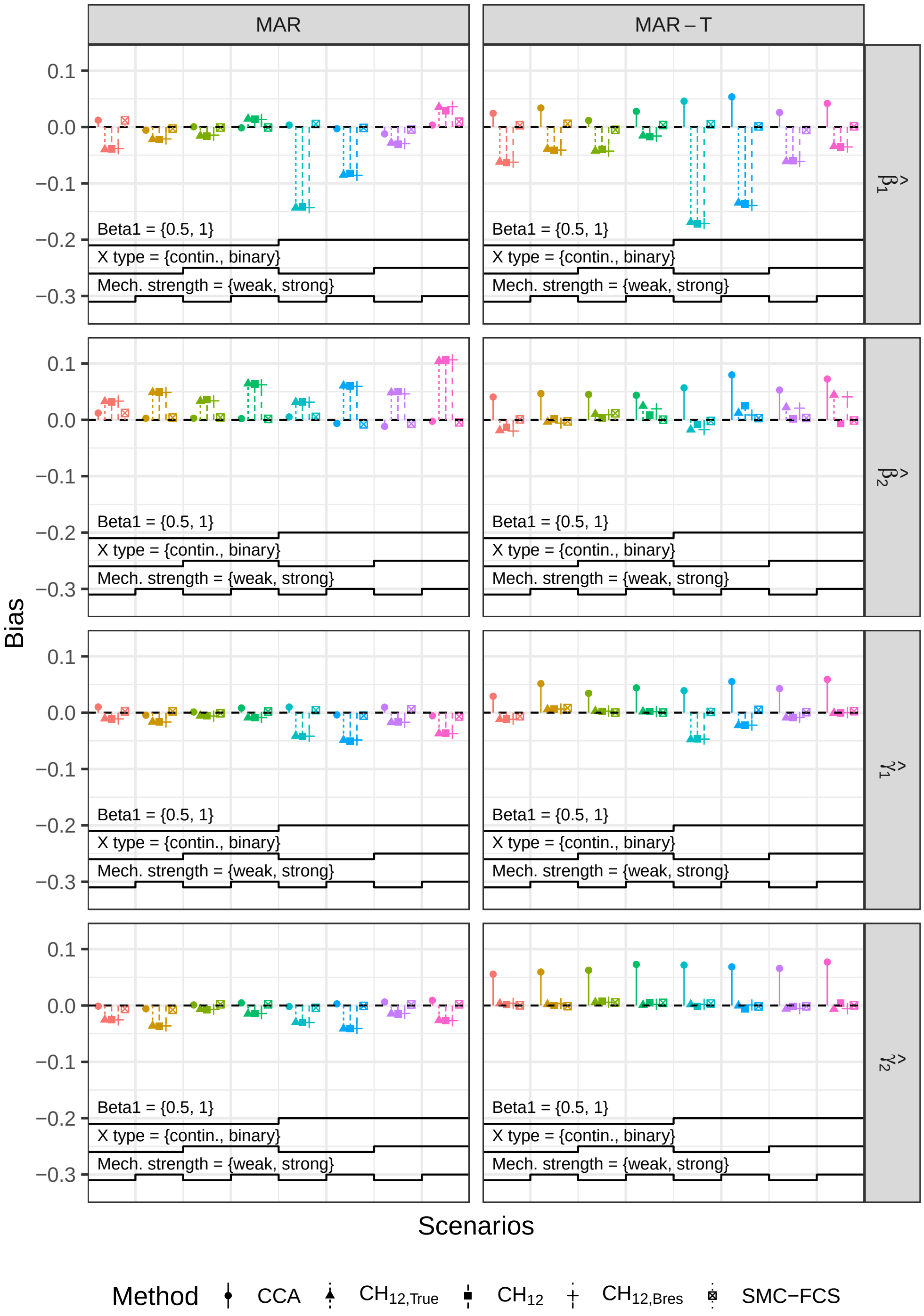

Figure 1: Summary of simulations assessing the Breslow estimator in the imputation model.

$$\operatorname{logit} P(X=1 \mid T,D,Z) = \log p(T,D \mid X=1,Z) - \log p(T,D \mid X=0,Z) + \operatorname{logit} P(X=1 \mid Z),$$
$$= \zeta_0 + \zeta_1 Z + \sum_{k=1}^{K} I(D=k)\beta_k - \sum_{k=1}^{K} H_{k0}(T)\exp(\gamma_k Z)(\mathrm{e}^{\beta_k}-1).$$

After approximating the $\exp(\gamma_k Z)$ terms (which currently make the right-hand side non-linear), we seemingly only need to assume $H_{k0}(T) \approx H_k(T)$, and we can then implement the imputation model using the Nelson-Aalen estimator. We argue that in reality, we should be thinking of $H_{k0}(T)\exp(\gamma_k Z) \approx H_k(T)$ rather than $H_{k0}(T) \approx H_k(T)$. Therefore, we see why CH_{12} should perhaps always be preferred to $CH_{12,\mathrm{Bres}}$ in practice: for small $\mathrm{Var}(\gamma_k Z)$ both will likely perform equivalently, while a larger $\mathrm{Var}(\gamma_k Z)$ may be better captured by the marginal cumulative hazards.

1.2 Setting with $K > 2$ competing events

The performance of both MICE and SMC-FCS has hitherto also not been investigated when there are more than two competing events. We focus here on $K = 3$ causes, and modify our data-generating mechanism (for event times) as follows

$$\begin{aligned}
\tilde{T}_1 &\sim \mathrm{Weibull}(\kappa_1, \lambda_1 = \lambda_{10} e^{\beta_1 X + \gamma_1 Z}),\\
\tilde{T}_2 &\sim \mathrm{Weibull}(\kappa_2, \lambda_2 = \lambda_{20} e^{\beta_2 X + \gamma_2 Z}),\\
\tilde{T}_3 &\sim \mathrm{Exp}(\lambda_3 = \lambda_{30} e^{\beta_3 X + \gamma_3 Z}),\\
C &\sim \mathrm{Exp}(\lambda_C),
\end{aligned}$$

where $\{\kappa_1, \lambda_{10}, \kappa_2, \lambda_{20}\} = \{1.5, 0.04, 0.53, 0.21\}$ are the Weibull parameters from the 'different' hazards condition in the two-cause setting, and for cause 3 we fix $\lambda_{30} = 0.1$. In terms of regression coefficients, we set $\{\beta_2, \beta_3, \gamma_1, \gamma_2, \gamma_3\} = \{0.5, 0.75, 1, 0.5, 0.25\}$, and vary β_1 as specified in Table 1. The independent censoring rate stays fixed as $\lambda_C = 0.14$.

We compared the following methods:

1. CC - an analysis run on a dataset after listwise deletion.

2. CH_{123} - MI with imputation model predictors including Z, the event indicator $D \in \{0,1,2,3\}$ as a four level factor variable, and the cumulative hazards for all events $\hat{H}_1(T)$, $\hat{H}_2(T)$ and $\hat{H}_3(T)$.

3. $CH_{123,\mathrm{Int}}$ - identical to CH_{123}, with the addition of the interactions $\hat{H}_1(T) \times Z$, $\hat{H}_2(T) \times Z$ and $\hat{H}_3(T) \times Z$.

4. smcfcs - the approach outlined in the manuscript, using Z as sole predictor in the $X \mid Z$ model (default setting).

The results are summarized in Figure 2. Finding are consistent with the two-cause setting: smcfcs was unbiased across all explored settings, CC was unbiased throughout the MAR setting. Concerning the MICE methods, there was some stronger evidence (relative to the two-cause setting) in favour of including the $\hat{H}_k(T) \times Z$ interaction terms in the imputation model, particularly in the estimation of β_2 and β_3.

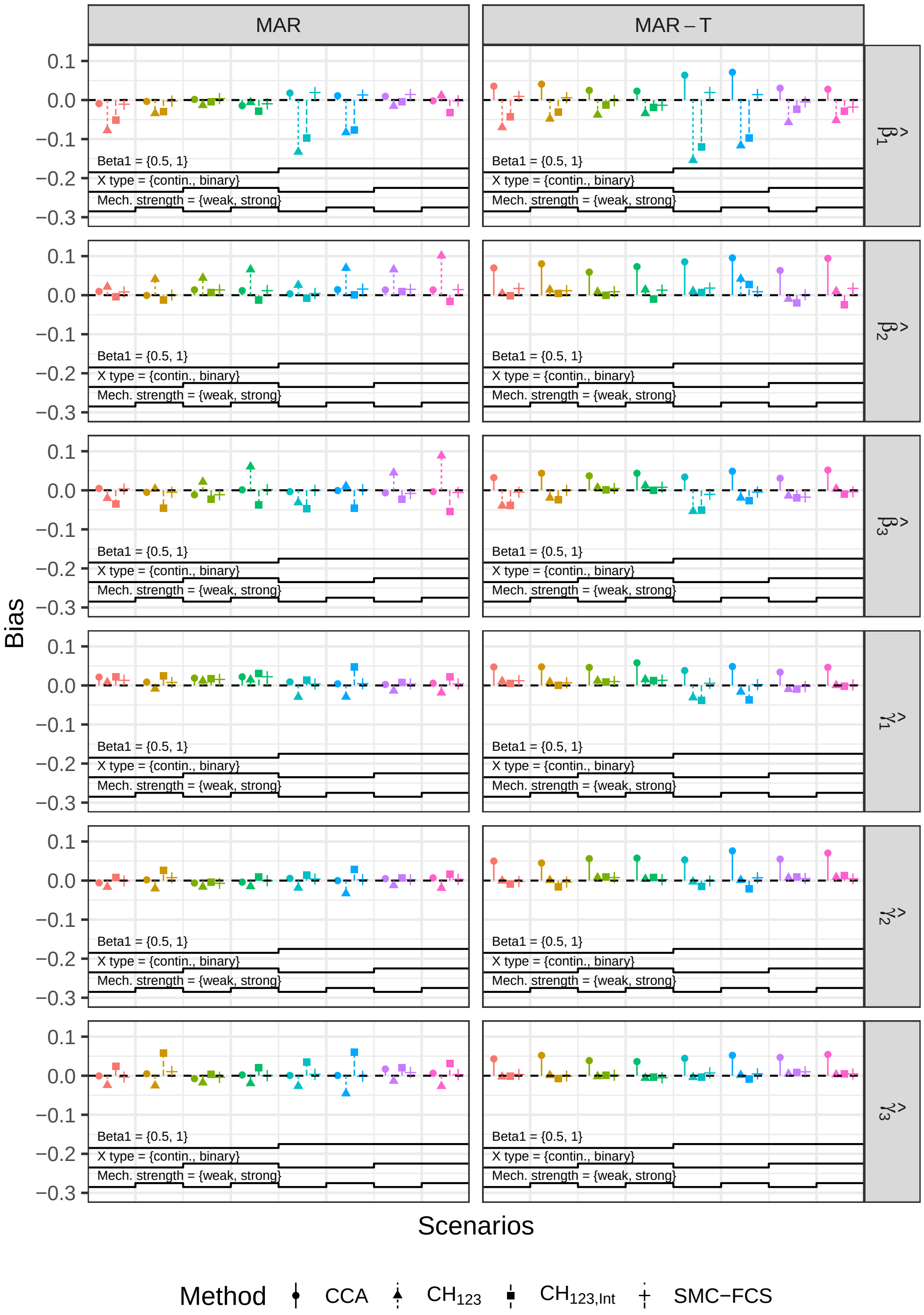

Figure 2: Summary of simulations assessing the setting with $K = 3$ competing events.

2 Cumulative incidence curves MDS long-term data

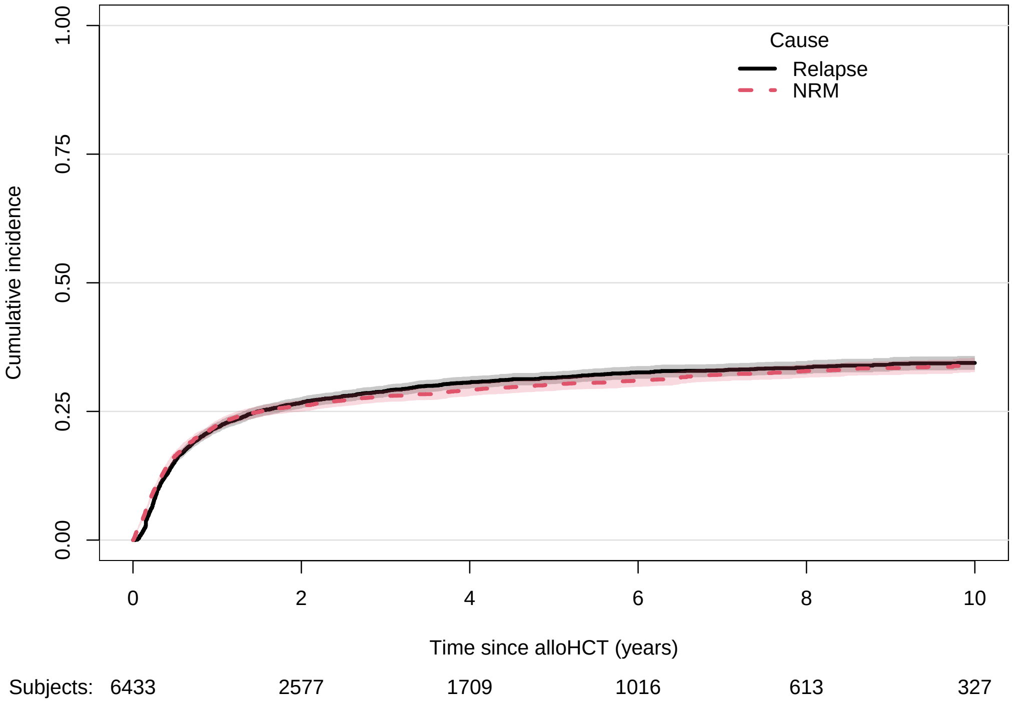

Figure 3: Non-parametric cumulative incidence curves for relapse and non-relapse mortality in the MDS long-term dataset. These formed the basis for the 'similar' baseline hazards parametrization in the simulation study.

3 Figures from supplementary material II referenced in manuscript

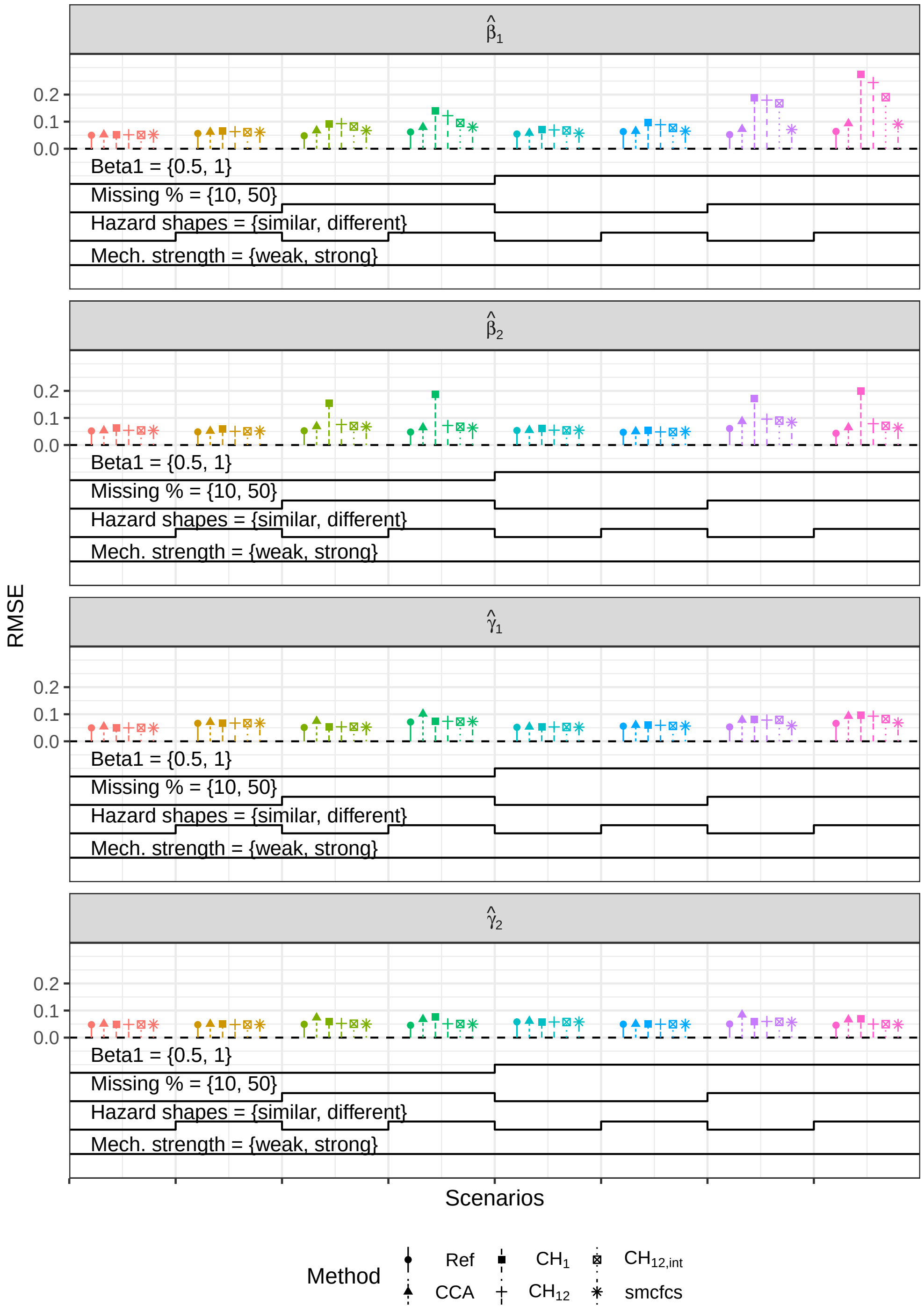

Figure 4: Figure 2.1.2 from supplementary material II, showing RMSE for regression coefficients under continuous X and MCAR mechanism.

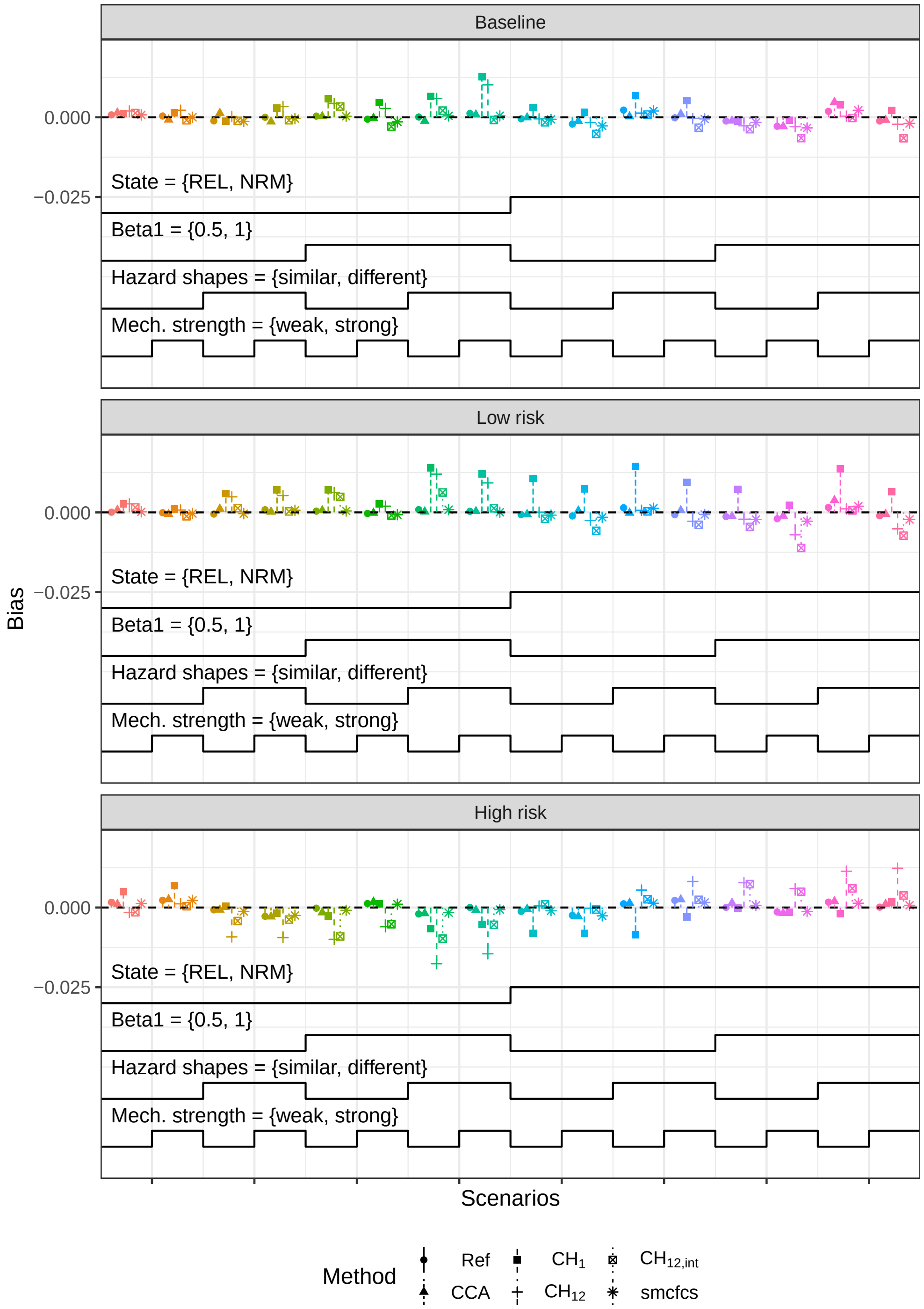

Figure 5: Figure 1.2.1 from supplementary material II; showing bias in predicted probabilities under continuous X and MAR mechanism.

References

[1] Ian R. White and Patrick Royston. Imputing missing covariate values for the Cox model. *Statistics in Medicine*, 28(15):1982–1998, 2009. ISSN 1097-0258.
